# Fighting *Staphylococcus aureus* infections with light and photoimmunoconjugates

**DOI:** 10.1172/jci.insight.139512

**Published:** 2020-11-19

**Authors:** Mafalda Bispo, Andrea Anaya-Sanchez, Sabrina Suhani, Elisa J. M. Raineri, Marina López-Álvarez, Marjolein Heuker, Wiktor Szymański, Francisco Romero Pastrana, Girbe Buist, Alexander R. Horswill, Kevin P. Francis, Gooitzen M. van Dam, Marleen van Oosten, Jan Maarten van Dijl

**Affiliations:** 1 Department of Medical Microbiology and; 2 Department of Radiology, Medical Imaging Center, University of Groningen, University Medical Center Groningen, Groningen, Netherlands.; 3 Stratingh Institute for Chemistry, University of Groningen, Groningen, Netherlands.; 4 Department of Immunology and Microbiology, University of Colorado Anschutz Medical Campus, Aurora, Colorado, USA.; 5 PerkinElmer, Hopkinton, Massachusetts, USA.; 6 Department of Surgery, Division of Surgical Oncology, Nuclear Medicine and Molecular Imaging, Intensive Care, University of Groningen, University Medical Center Groningen, Groningen, Netherlands

**Keywords:** Microbiology, Therapeutics, Bacterial infections, Drug therapy, Immunoglobulins

## Abstract

Infections caused by multidrug-resistant *Staphylococcus aureus*, especially methicillin-resistant *S. aureus* (MRSA), are responsible for high mortality and morbidity worldwide. Resistant lineages were previously confined to hospitals but are now also causing infections among healthy individuals in the community. It is therefore imperative to explore therapeutic avenues that are less prone to raise drug resistance compared with today’s antibiotics. An opportunity to achieve this ambitious goal could be provided by targeted antimicrobial photodynamic therapy (aPDT), which relies on the combination of a bacteria-specific targeting agent and light-induced generation of ROS by an appropriate photosensitizer. Here, we conjugated the near-infrared photosensitizer IRDye700DX to a fully human mAb, specific for the invariantly expressed staphylococcal antigen immunodominant staphylococcal antigen A (IsaA). The resulting immunoconjugate 1D9-700DX was characterized biochemically and in preclinical infection models. As demonstrated in vitro, in vivo, and in a human postmortem orthopedic implant infection model, targeted aPDT with 1D9-700DX is highly effective. Importantly, combined with the nontoxic aPDT-enhancing agent potassium iodide, 1D9-700DX overcomes the antioxidant properties of human plasma and fully eradicates high titers of MRSA. We show that the developed immunoconjugate 1D9-700DX targets MRSA and kills it upon illumination with red light, without causing collateral damage to human cells.

## Abstract 2

An immunoconjugate for targeted photodynamic therapy of Staphylococcus aureus infections kills MRSA with high efficacy upon illumination with red light.

## Graphical abstract

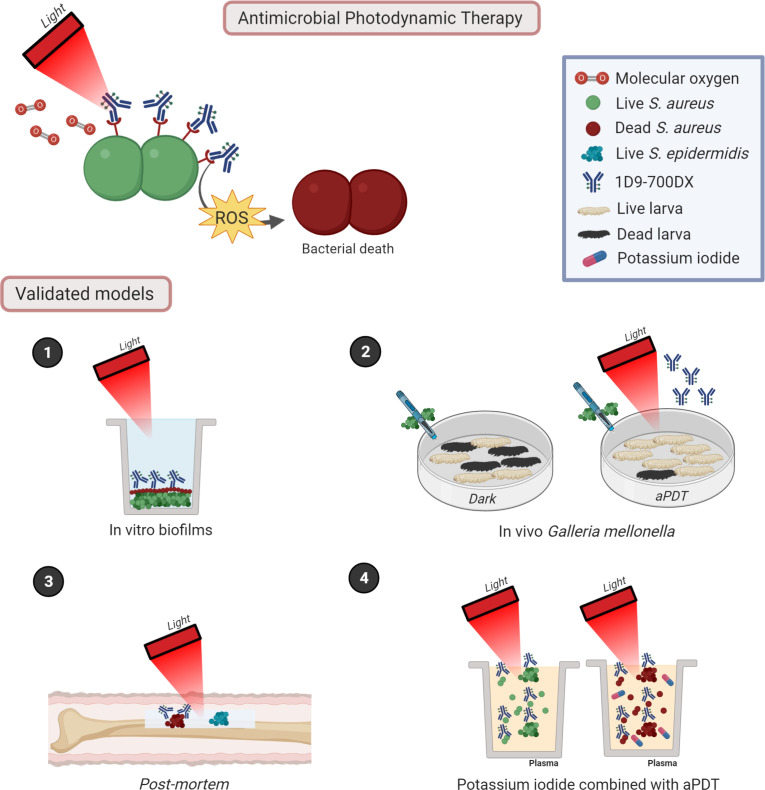

## Introduction

*Staphylococcus aureus* is a Gram-positive bacterium, part of the normal human microbiota, but also a leading cause of bacteremia, endocarditis, osteomyelitis, and skin and soft tissue infections (1). Such infections are increasingly difficult to treat, partly due to acquired antibiotic resistances, as exemplified by methicillin-resistant *S*. *aureus* (MRSA) (2, 3) and partly due to the bacterium’s ability to form thick biofilms on tissues and implanted biomaterials (e.g., catheters, heart valves, and prosthetic joints) (4). The staphylococcal biofilms are, in general, multilayered structures of bacteria embedded in a matrix of proteins, DNA, and host components, which protect the bacteria against the host immune system and most antimicrobial agents (5). Therefore, there is a major need to explore alternative antistaphylococcal therapies to complement or enhance the currently available therapies.

A potential alternative therapeutic approach is antimicrobial photodynamic therapy (aPDT) (6), an emerging treatment modality based on the photoactivation of a photosensitizer with light of an appropriate wavelength. Together with molecular oxygen, the photosensitizer will generate ROS, mostly singlet oxygen (^1^O_2_), that kill the infecting organism. Moreover, bacterial resistance to photosensitizers has not yet been reported. An ideal photosensitizer should be selectively toxic to the pathogen with minimal damage to the host. In addition, the photosensitizer should have a large absorption coefficient in the visible spectrum, especially in the long wavelength (near-infrared) region, to allow effective penetration of light into the infected tissue (7). Recently, the development of more effective antibacterial aPDT agents has been accomplished by conjugating second-generation photosensitizers with biomolecular recognition motifs, such as carbohydrates, bacteriophages, or monoclonal antibodies (8–12). However, there are currently only a few reports on the development of targeted aPDT against *S*. *aureus*, and none were tested in vivo.

mAbs against bacterial surface proteins seem to be ideal carriers for photosensitizers since, by binding their cognate antigens, they improve distribution and concentration of the drug at the site of injury/disease. This will keep collateral damage to host cells to a minimum (9). A few anti-staphylococcal mAbs have been developed and evaluated in late-stage clinical studies (13), but their efficacy in passive immunization was insufficient. It is, however, well conceivable that the efficacy of anti-staphylococcal mAbs can be enhanced by conjugation to photosensitizers. We have previously developed a fully human mAb — 1D9 — which targets the immunodominant staphylococcal antigen A (IsaA), a strictly conserved noncovalently cell wall–bound lytic transglycosylase that is exposed on the surface of all *S*. *aureus* isolates tested so far (14–18). 1D9 was preclinically shown to be highly effective in the noninvasive diagnosis of *S*. *aureus* soft tissue infections, as well as spinal and shoulder implant infections in mice using PET-CT or in vivo fluorescence imaging (19–21). Importantly, this diagnostic approach offers the advantage that it can distinguish infection from aseptic inflammation because it directly targets the causative pathogen. Accordingly, the fluorescently labeled 1D9 was shown to facilitate intraoperative image–guided selective debridement of *S*. *aureus* biofilms on spinal and shoulder implants (20, 21).

Building on the observation that 1D9 can be applied for targeted infection imaging and image-guided surgery, we envisaged that this mAb could be applicable as a targeting agent for aPDT to treat *S*. *aureus* infections. The added value of a 1D9-fluorochrome conjugate with photosensitizer activity would be that it may be applied as a so-called theranostic agent, allowing both the direct diagnosis of infection and targeted in situ antimicrobial therapy. In the present study, 1D9 was therefore conjugated with IRDye700DX, a commercially available silicon phthalocyanine derivative with relatively hydrophilic properties and a strong absorption band in the near-infrared region of the spectrum (22). Furthermore, this potent photosensitizer can be readily conjugated to proteins and has been widely explored for PDT of cancer (23–26). The here presented study demonstrates the feasibility of applying a 1D9-IRDye700DX immunoconjugate (hereafter referred to as 1D9-700DX) as an innovative targeted aPDT agent that disrupts *S*. *aureus* biofilms in vitro, rescues larvae of the wax moth *Galleria mellonella* from MRSA infections, and is effective in *S*. *aureus* killing in a human postmortem infection model. Importantly, when combined with the enhancing agent potassium iodide (KI), the 1D9-700DX immunoconjugate can fully overcome potent antioxidant defenses of the human body, allowing the complete eradication of high titers of MRSA.

## Results

### In vitro characterization of 1D9-700DX.

Conjugation of 1D9 to the IRDye700DX *N*-hydroxysuccinimide (NHS) ester, to obtain 1D9-700DX, was achieved through reaction of the NHS ester with the primary amines of lysine residues in the mAb. Judged by the absorbance values at 280 nm (protein) and 690 nm (IRDye700DX), this resulted in approximately 1–3 conjugated dye molecules per mAb molecule. MALDI-TOF mass spectrometry was used to determine the exact mass of the mAb 1D9 — 148,503 *m/z* — and to visualize the shift toward a higher *m/z* of the 1D9-700DX immunoconjugate — 152,110 *m/z* — corresponding to approximately 3 dye molecules per mAb (Supplemental Figure 1A; supplemental material available online with this article; https://doi.org/10.1172/jci.insight.139512DS1). Furthermore, the covalent association between the mAb and IRDye700DX was evidenced by LDS-PAGE of 1D9-700DX under reducing conditions, where the separated heavy (~50 kDa) and light (~25 kDa) chains were both shown to fluoresce (Supplemental Figure 1B). Moreover, we analyzed the in vitro immunoreactivity of 1D9-700DX with an ELISA (Supplemental Figure 1C). This showed that, with 1D9-700DX, 94.3% ± 2.6% (mean ± SEM) of binding was achieved, which is identical to the immunoreactivity of the unconjugated mAb 1D9 (95.1% ± 3.4%).

Photobleaching of photosensitizers is caused by oxidative photodegradation due to reaction with self-generated ^1^O_2_. Thus, it can be identified by a decrease of the bands in the absorption spectra without formation of new peaks (27). Aliquots of 1D9-700DX were irradiated for several periods of time using a high-output LED device that emits light at 690 nm (28), and the immunoconjugate was found to be photostable until 5 minutes of exposure at 100 mW.cm^–2^ (30 J.cm^–2^) (Supplemental Figure 1D). Of note, the maximum absorbance peak of IRDye700DX is at 689 nm. Thus, to achieve the highest efficiency in ROS production, one should irradiate at the λ_max_ of this molecule, since the attenuation coefficient at that wavelength is the largest, and therefore the ROS generation efficiency is maximal. The ability of 1D9-700DX to produce ^1^O_2_ was indirectly evaluated in DMSO, using 1,3-diphenylisobenzofuran (DPBF), an established ^1^O_2_ scavenger (Supplemental Figure 1E) (29). 1D9-700DX was able to photo-oxidize 91% of the initial DPBF, which was present at a 100:1 molar ratio compared with the photosensitizer, within 15 seconds exposure to red light. This demonstrated that the immunoconjugate had retained a high capability to produce ^1^O_2_.

### 1D9 binding by S.

**Figure 1:**
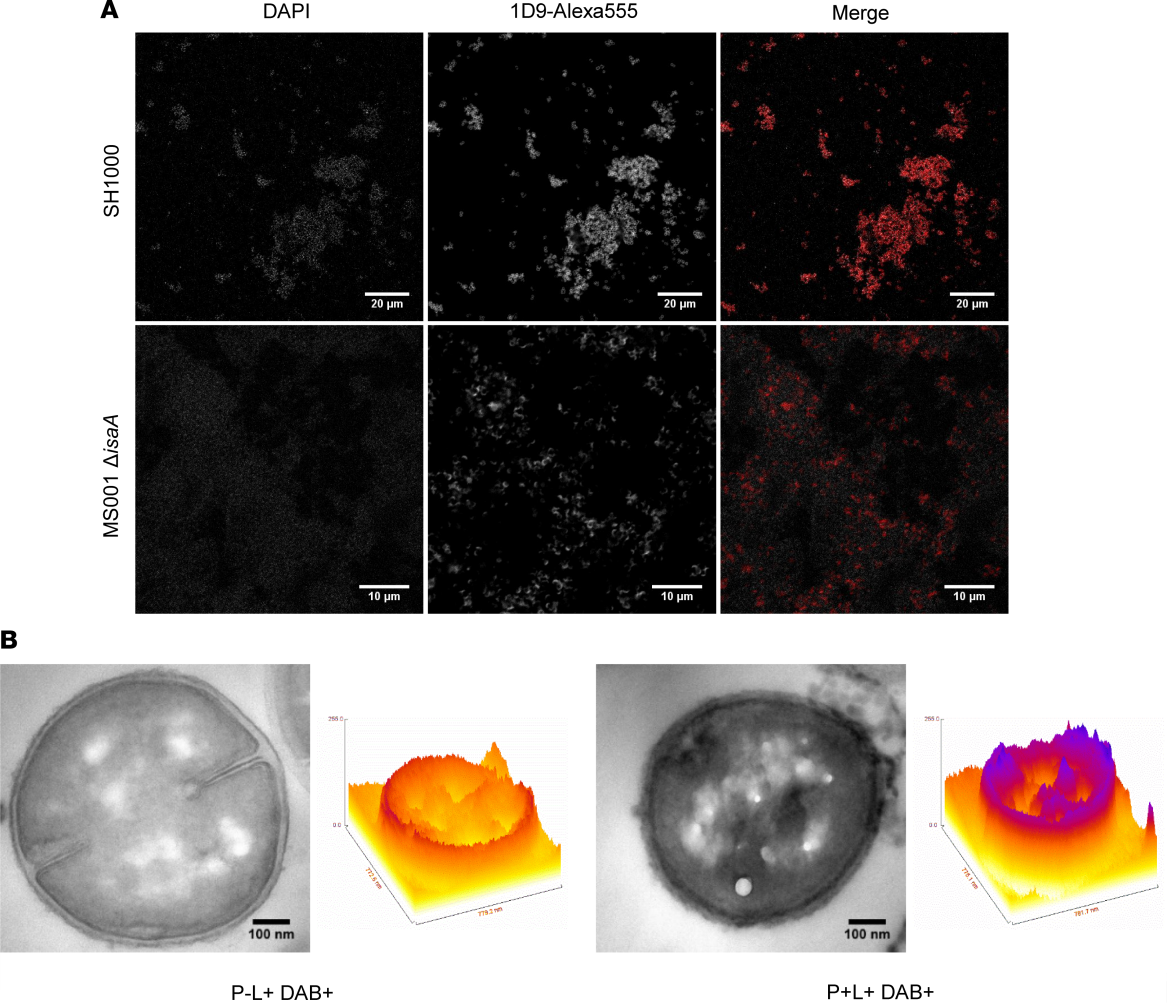
IsaA-specific targeting of 1D9-based immunoconjugates to the *S.* *aureus* cell surface. (**A**) Colocalization of the 1D9-Alexa555 (red) immunoconjugate with *S*. *aureus* SH1000 and MS001 Δ*isaA* stained with DAPI (gray). A total of 3 μg.mL^–1^ of 1D9-Alexa555 was incubated with diluted bacterial overnight cultures (OD_600_ = 1) and imaged with a confocal laser scanning microscope. (**B**) ROS production and localization of 1D9-700DX in *S*. *aureus* Newman WT determined by DAB photooxidation. Both samples contained 1 mg.mL^–1^ DAB (DAB^+^) and were irradiated with red light (60 J.cm^–2^; L^+^) for 10 minutes at 100 mW.cm^–2^ prior fixation. Samples were supplemented with 1.3 μM of the 1D9-700DX photosensitizer (P^+^) or PBS (P^–^). Images were recorded by TEM. Intensity surface plots were created using FIRE LUT and the surface plot tool in ImageJ.

*aureus*. The mAb 1D9 binds IsaA with high affinity, but like any human IgG1, it also binds to the 2 immunoglobulin-binding proteins SpA and Sbi of *S*. *aureus* (20, 30, 31). Therefore, we compared by fluorescence microscopy the binding of this mAb to IsaA and SpA/Sbi. To this end, 2 WT *S*. *aureus* strains (SH1000 and Newman), and the respective isogenic deletion mutants lacking IsaA (MS001) or SpA and Sbi (Newman Δ*spA*, Δ*sbi*) were incubated with 1D9 that had been labeled with Alexa Fluor 555 (1D9-Alexa555). The 1D9-Alexa555 immunoconjugate readily detected all the strains, but the detectable binding was notably reduced in the IsaA-deficient strain (Figure 1A). In contrast, compared with the respective WT strain Newman, binding was barely reduced in the Newman Δ*spA*, Δ*sbi* mutant (Supplemental Figure 2). Thus, under the tested conditions, the 1D9 mAb binds to IsaA with minimal background binding to the SpA/Sbi proteins on the *S*. *aureus* surface.

To visualize the localization and photoactivity of 1D9-700DX upon binding to *S*. *aureus* cells, DAB photooxidation on the bacterial cell surface was assessed by transmission electron microscopy (TEM) (32, 33). In this approach, ROS-generating species can be localized with high resolution by detecting the ROS-induced polymerization of DAB. The DAB polymer’s osmiophilicity will increase osmium staining at the site where ROS production takes place, which is detectable by TEM. Since the lifetime of ROS is very short, we can correlate the site of DAB polymer formation with the location of the ROS-producing molecule. Figure 1B shows that, indeed, 1D9-700DX produces ROS at the bacterial cell surface, as enhanced osmium staining is only observed for the cells that were incubated with 1D9-700DX and treated with red light at a radiance exposure of 60 J.cm^–2^ (P^+^L^+^ DAB^+^). These experiments, therefore, highlight the ability of 1D9-700DX to produce ^1^O_2_ at the bacterial cell surface, underlining its applicability for targeted aPDT.

### In vitro killing of S.

**Figure 2:**
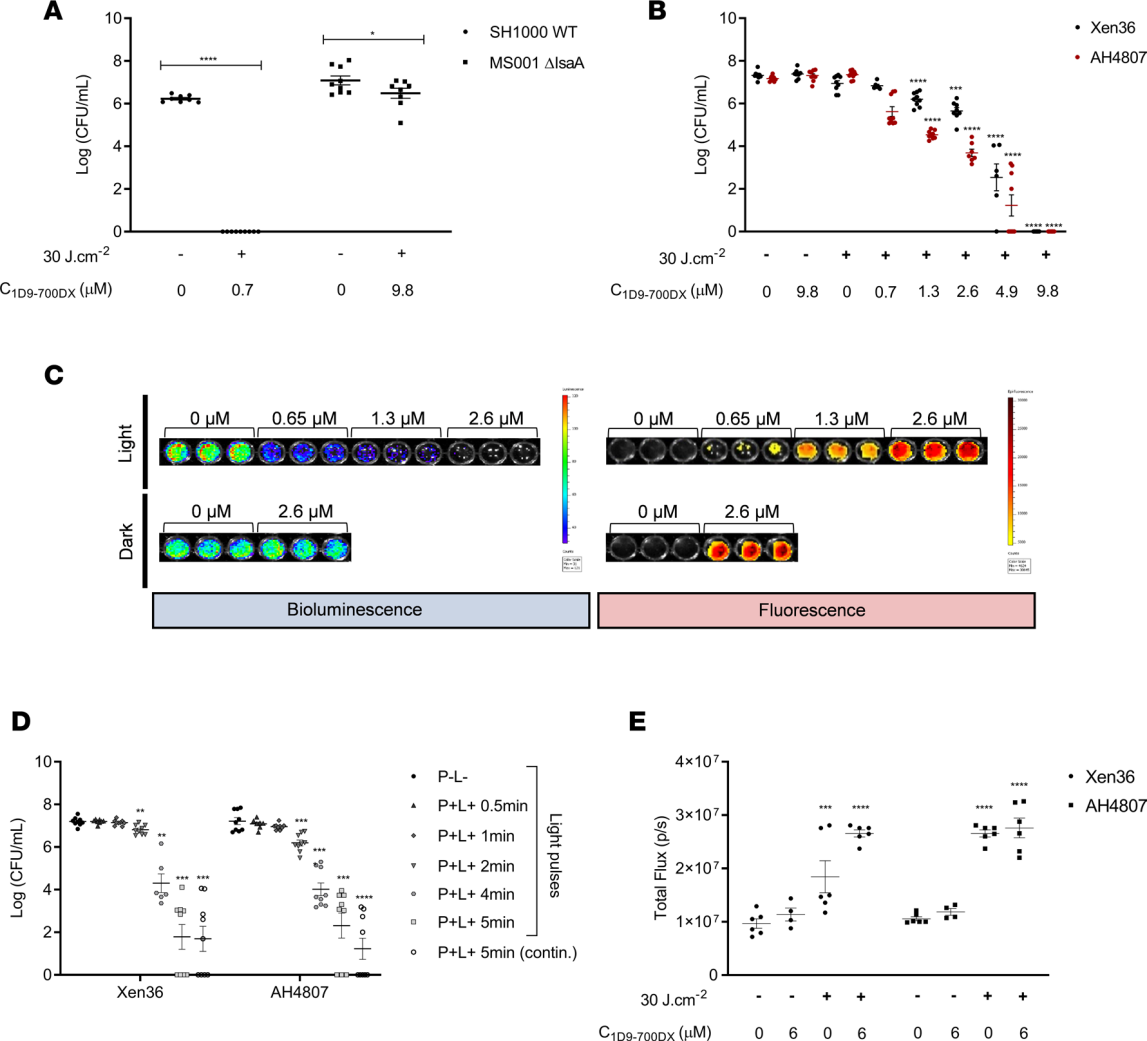
Photo-activated killing of *S. aureus* by 1D9-700DX. (**A** and **B**) Photo-activated killing of *S*. *aureus* SH1000 WT versus MS001 Δ*isaA* (**A**) or Xen36 versus CA-MRSA AH4807 (**B**) grown to exponential phase (~1 × 10^7^ CFU/mL) upon treatment with 1D9-700DX or without photosensitizer (**A**), or step-wise increasing concentrations of 1D9-700DX (0.7–9.8 μM) (**B**). Bacteria were irradiated with red light at a radiant exposure of 30 J.cm^–2^ (+) or kept in the dark (–). (**C**) Bacterial bioluminescence (open emission filter, 10-second exposure) and fluorescence of 1D9-700DX (emission filter, Cy5.5; excitation, 640 nm; 10-second exposure) recorded with the IVIS Lumina II upon aPDT of *S*. *aureus* Xen36 or AH4807 (~1 × 10^8^ CFU/mL) with increasing concentrations of 1D9-700DX (0–2.6 μM). (**D**) Red light dose-response analysis of the killing of *S*. *aureus* Xen36 or CA-MRSA AH4807 grown to exponential phase and subjected to aPDT with 4.9 μM of 1D9-700DX or without photosensitizer. Bacteria were irradiated with red light for different periods of time (0.5–5 minutes) or kept in the dark (–). As a control, the bacteria were subjected to continuous (contin.) red light irradiation for 5 minutes. (**E**) H_2_O_2_ production upon aPDT of *S*. *aureus* Xen36 and AH4807 with 6 μM of 1D9-700DX or without photosensitizer. H_2_O_2_ was detected with 10 μM of an AquaSpark Peroxide Probe. In all experiments, irradiation was performed with a LED system that emits red light. Data are presented as mean ± SEM of 3 experiments performed in triplicates. Two-way ANOVA with subsequent Šidák multiple-comparison tests were used for statistical analysis. Significant differences compared with the negative control group (no 1D9-700DX and no light) are marked as follows: *P < 0.03; **P < 0.002; ***P < 0.0002; ****P < 0.0001.

*aureus by photoactivated 1D9-700DX*. To study the in vitro antimicrobial activity of 1D9-700DX, we first investigated the survival of WT or IsaA-deficient *S*. *aureus* incubated with or without the immunoconjugate in the absence or presence of red light (Figure 2A). A total of 1 × 10^7^ CFU of *S*. *aureus* SH1000 and the isogenic IsaA-deficient mutant MS001 were incubated with 0.7 and 9.8 μM of 1D9-700DX, respectively. The bacteria were then irradiated with 30 J.cm^–2^ of red light, which is the maximum radiant exposure that does not cause photobleaching of IRdye700DX (Supplemental Figure 1D). After CFU counting, the results showed that 0.7 μM of 1D9-700DX completely eradicated the SH1000 WT strain, while 9.8 μM decreased the viability of the MS001 strain that lacks IsaA by less than 1 log. This showed that 1D9 binding to the SpA/Sbi proteins is contributing only to a minor extent to the aPDT efficacy, arguing that IsaA-specific targeted aPDT is superior over the intrinsic IgG1-binding capabilities of SpA/Sbi.

To obtain more detailed insights into the photoactivated killing of *S*. *aureus* upon exposure to 1D9-700DX, we incubated the methicillin-sensitive *S*. *aureus* (MSSA) Xen36 strain and the MRSA strain AH4807 with different concentrations of the immunoconjugate in the presence or absence of red light. The advantage of using these strains is that they have been genetically engineered to become bioluminescent through the expression of modified *lux* genes derived from *Photorhabdus luminescens* (Supplemental Table 1). As shown in Figure 2B, the bacterial viability only decreased upon exposure to 1D9-700DX and red light, whereas no effect on viability was observed in the absence of the immunoconjugate or red light. Furthermore, the Xen36 MSSA strain appeared to be somewhat more resilient to aPDT with 1D9-700DX compared with the AH4807 MRSA strain. A 1D9-700DX concentration of 9.8 μM was sufficient to completely eradicate 1 × 10^7^ CFU/mL of both strains. The different susceptibility of the 2 *S*. *aureus* strains is most likely related to the fact that the Xen36 strain expresses lower levels of the IsaA target protein and the IgG-binding proteins SpA/Sbi than the AH4807 strain, as shown by a Western blotting with 1D9 conjugated to IRDye800CW (Supplemental Figure 3, A and B). Conversely, the *S*. *aureus* strain SH1000 expressed the highest levels of IsaA and SpA/Sbi, rendering it more sensitive to 1D9-700DX after light activation (Figure 2A). The effect of aPDT with 1D9-700DX on the bacterial bioluminescence was also visualized using an In Vivo Imaging System (IVIS Lumina II). As exemplified in Figure 2C, increasing immunoconjugate concentrations correlated well with decreasing bioluminescence of the AH4807 strain. Of note, bioluminescence was no longer detectable at a 1D9-700DX concentration of 2.6 μM, suggesting that most bacteria were already deenergized at this concentration of the immunoconjugate, whereas higher concentrations — 9.8 μM — are required to achieve 100% killing (Figure 2B).

After determining the minimal 1D9-700DX concentration to eliminate 1 × 10^7^ CFU/mL of the MSSA and MRSA strains, we tested whether different times of irradiation with red light at 100 mW.cm^–2^ would influence the bacterial killing when compared with continuous irradiation at 30 J.cm^–2^, as used in the above experiments (Figure 2D). After incubation with 4.9 μM of 1D9-700DX, the Xen36 and AH4807 strains were irradiated with red light. Already after 2 minutes of irradiation, a significant decrease of the bacterial viability was observed. Nevertheless, there was a significant difference detectable upon 2- or 5-minute irradiation for both strains, which implies that 30 J.cm^–2^ is an optimal radiant exposure for bacterial killing, without compromising the properties of the photosensitizer.

Next, the importance of conjugating 1D9 mAb to IRDye700DX for targeted aPDT was evaluated by comparing the efficacy of 1D9-700DX to that of the IRDye700DX carboxylate. Of note, the IRDye700DX carboxylate will not bind to *S*. *aureus* because it lacks the NHS ester that was used to cross-link the IRDye700DX to the 1D9 antibody (Supplemental Figure 4). The IRDye700DX carboxylate, thus, mimics a small-molecule photosensitizer without target specificity. To compare the efficacy of 1D9-700DX and IRDye700DX carboxylate, we incubated 1 × 10^9^ CFU/mL of *S*. *aureus* AH4807 with 9.8 μM of each compound for 30 minutes, and we subsequently washed the bacteria with PBS to remove the unbound compounds. As shown in Supplemental Figure 5A, fluorescence imaging revealed ~2-fold higher binding of 1D9-700DX to the bacteria compared with the IRDye700DX carboxylate. Subsequently, the bacteria were irradiated with 30 J.cm^–2^ of red light, and their viability was assessed by CFU counting. This showed that treatment with 1D9-700DX resulted in a 2.5-log reduction of viable bacteria, while treatment with the IRDye700DX carboxylate did not affect the bacterial viability (Supplemental Figure 5B). This observation shows that targeted aPDT with 1D9-700DX is more effective than nontargeted aPDT.

Lastly, the production of ROS after aPDT with 1D9-700DX against *S*. *aureus* was determined using a dioxetane-based substrate for the detection of hydrogen peroxide (H_2_O_2_) and other peroxides in biological samples by luminescence. As shown in Figure 2E, there was significantly higher production of H_2_O_2_ in the irradiated sample groups compared with the ones that were kept in the dark. Of note, exposure to red light alone at 30 J.cm^–2^ is not toxic for the bacteria, as presented in Figure 2, B and C; as shown in Figure 1B, other ROS species, such as ^1^O_2_, are also produced.

### S. aureus biofilm destruction by photoactivated 1D9-700DX.

**Figure 3:**
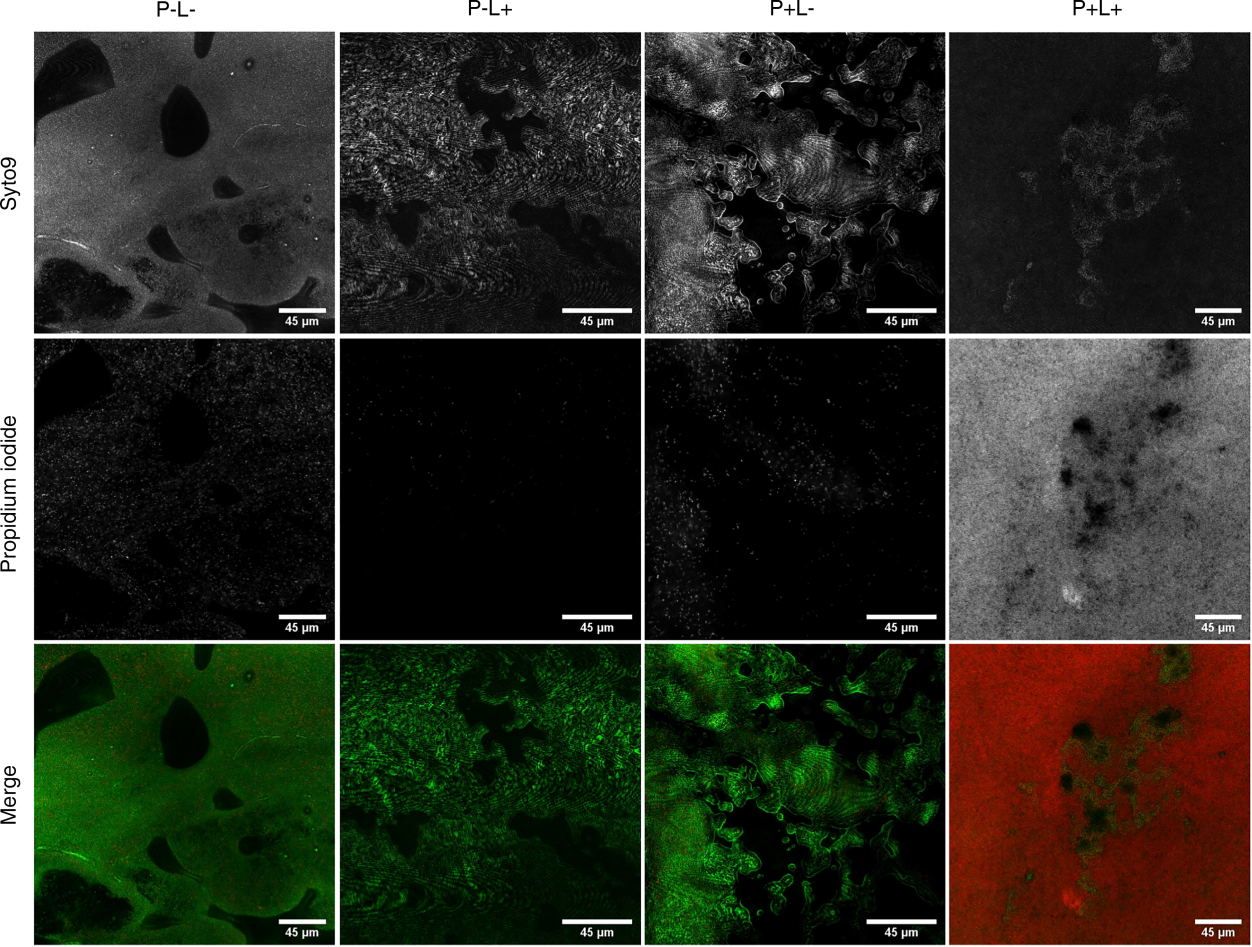
Confocal laser scanner microscopy images of biofilm formed by *S. aureus* NCTC8325-4. P^–^L^–^ show negative control (no photosensitizer [P] and no light [L]); P^–^L^+^ show the effect of light only; P^+^L^–^ show the effect of only the photosensitizer; and P^+^L^+^ show aPDT with photosensitizer and light. The biofilms were either incubated with 15.5 μM of 1D9-700DX or PBS, and they were either kept in the dark or treated with red light LEDs at a radiant exposure of 30 J.cm^–2^. To assess the bacterial viability, biofilms were stained with the BacLight Live/Dead stain. Green fluorescence (Syto9) marks living bacteria, and red fluorescence (propidium iodide) marks dead bacteria. Scale bars: 45 μm. Three-dimensional reconstructions from stacks of 2-dimensional confocal microscopy images recorded upon aPDT with 1D9-700DX (P^+^L^+^) is presented as Supplemental Video 1.

In an attempt to visualize the impact of aPDT with 1D9-700DX on biofilms, biofilms of *S*. *aureus* NCTC8325-4 were grown on coverslips and subjected to aPDT. Subsequently, LIVE/DEAD staining was performed with the dyes Syto9 and propidium iodide to, respectively, visualize live (green) and dead (red) bacteria by confocal laser scanning microscopy. Of note, some parts of the biofilm contain thicker layers, which translates into a brighter fluorescent signal and different biofilm phenotypes. Importantly, Figure 3 shows that aPDT with 15.5 μM of 1D9-700DX leads to substantial killing of bacteria in the biofilm. As shown in the respective *Z*-stacks (Supplemental Video 1), the aPDT with 1D9-700DX is particularly effective in killing bacteria in the outer layer of the biofilm, but bacteria in deeper layers of the biofilm are also targeted, albeit with a lower efficiency.

### In vivo aPDT in a G. mellonella infection model.

To assess the efficacy of aPDT with 1D9-700DX with clinical *S*. *aureus* isolates, we employed a *G. mellonella* larval infection model. Of note, the larvae of this wax moth represent a suitable alternative to mammalian infection models thanks to the fact that, in particular, the innate immune cells of larvae and mammals have comparable protective activities against invading bacteria (34). To establish the appropriate experimental conditions, we first infected *G*. *mellonella* larvae with community-associated (CA) or hospital-acquired (HA) MRSA isolates of the USA300 lineage (35) to assess their virulence. Specifically, different inocula were prepared to inject the larvae, using bacteria grown to midexponential or early-stationary phase on RPMI. As expected, increased numbers of injected bacteria resulted in increased mortality of the infected larvae (Supplemental Figure 6, A and B). When comparing inocula prepared from midexponential (Supplemental Figure 6A) or early-stationary phase bacteria (Supplemental Figure 6B), an increased virulence was observed for the latter, which was more pronounced at higher bacterial loads. This probably reflects increased synthesis of virulence factors upon entry into the stationary growth phase by the bacteria (36). Together, these experiments show that inocula containing 1 × 10^6^ CFU of the bacteria/larva in midexponential phase, or 1 × 10^5^ CFU/larva in the early-stationary phase, will kill 50% of the larvae in about 72 hours after infection, irrespective of their CA or HA origin (Supplemental Figure 6, A and B).

After defining the optimal conditions for infecting *G*. *mellonella* larvae with MRSA, we investigated suitable conditions to assess the in vivo efficacy of aPDT with 1D9-700DX. First, potentially toxic effects of aPDT with 1D9-700DX were evaluated in uninfected larvae. To this end, uninfected larvae were injected with 40 mg.kg^–1^ of 1D9-700DX (i.e., a comparable dose as in the biofilm experiments in Figure 3) and exposed to different doses of red light (0–120 J.cm^–2^). The survival rate of the larvae was assessed 72 hours after irradiation. The highest dose for which no larval death, melanization (an indicator of high larval morbidity), or other indicators of toxic effects (e.g., impaired motility) were observed was reached after 45 seconds of irradiation at 100 mW.cm^–2^ (4.5 J.cm^–2^) (Supplemental Figure 6C). Additionally, we verified that injection of 1D9-700DX at 40 mg.kg^–1^ with or without light exposure, or mock treatment with PBS, was not toxic for the larvae (Supplemental Figure 6D).

**Figure 4:**
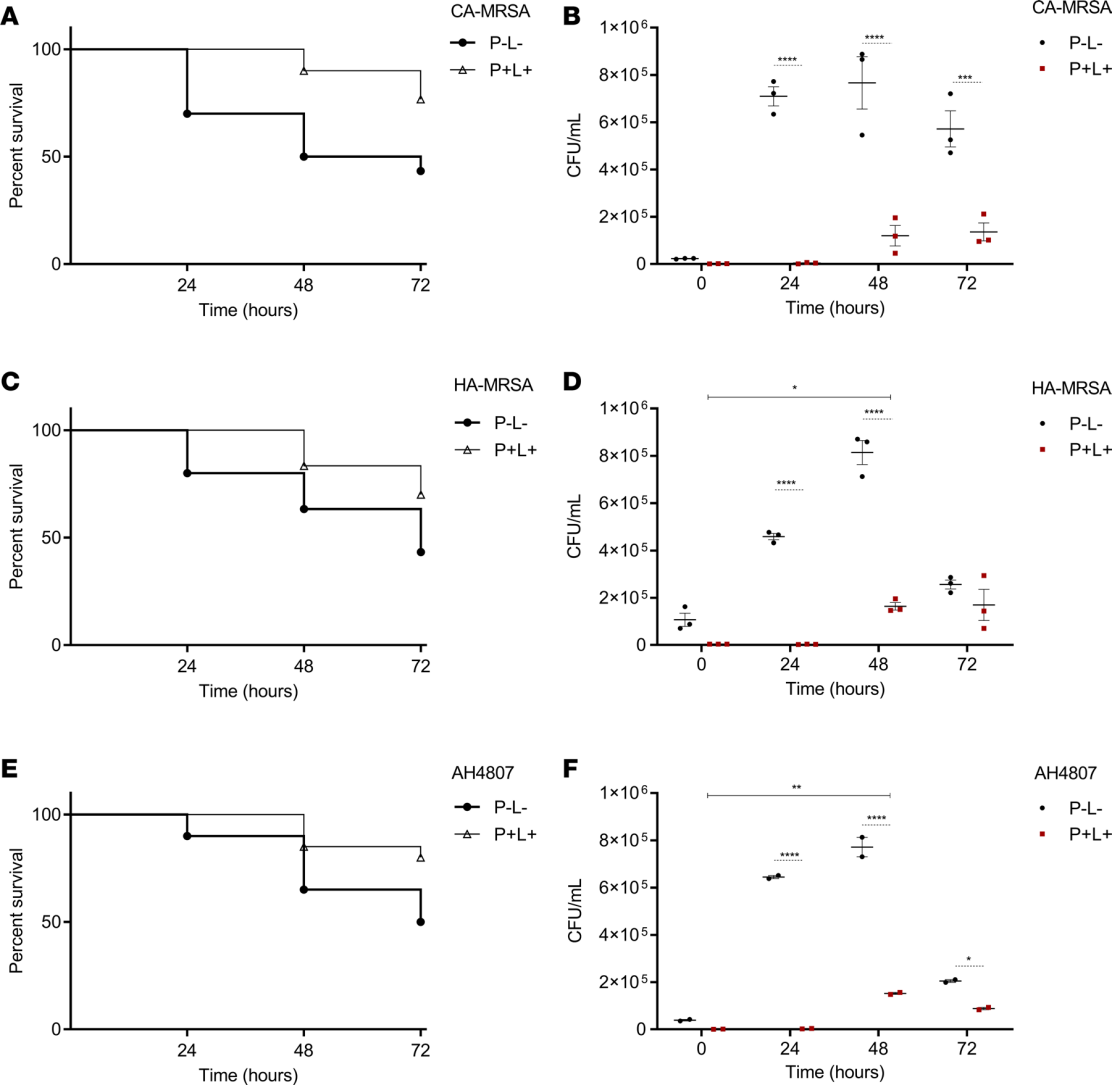
aPDT of *S. aureus* infection in G. mellonella. Larvae were infected with CA-MRSA USA300 D15-GFP, HA-MRSA USA300 D17-GFP, or CA-MRSA AH4807 grown to midexponential phase. For the P^+^L^+^ groups, 40 mg.kg^–1^ of 1D9-700DX (P^+^) were injected in the *G*. *mellonella* larvae at 90 minutes after bacterial inoculation. Thirty minutes after injection of the photosensitizer, red light treatment (L^+^) was performed at a radiant exposure of 4.5 J.cm^–2^ (45 seconds irradiation, 100 mW.cm^–2^). The P^–^L^–^ group neither received the immunoconjugate nor light exposure. (**A**, **C**, and **E**) *G*. *mellonella* survival was monitored at 24, 48, and 72 hours after treatment. (**B**, **D**, and **F**) Persistence of *S*. *aureus* in the hemolymph. Bacterial quantification (CFU/mL) in the hemolymph of 3 surviving larvae was determined at 0, 24, 48, and 72 hours after treatment. Data are presented as mean ± SEM of 2 (**E** and **F**) and 3 (**A**–**D**) independent experiments with groups of 10 larvae (*n* = 10). Gehan-Breslow-Wilcoxon and 2-way ANOVA tests with subsequent Šidák multiple-comparison tests were used for statistical analysis of survival curves and bacterial persistence in the larvae hemolymph, respectively. Significant differences compared with the negative control group (P^–^L^–^) are marked as follows: *P < 0.03; **P < 0.002; ***P < 0.0002; ****P < 0.0001.

For the aPDT experiments with infected larvae, we first established dose-response curves using different concentrations of 1D9-700DX to determine whether concentrations lower than 40 mg.kg^–1^ can be applied. Indeed, aPDT with 1D9-700DX was able to rescue CA- or HA-MRSA–infected larvae, with the best survival rates at 72 hours after irradiation being observed at a dose of 40 mg.kg^–1^ (Supplemental Figure 6E). Next, we applied the established optimal conditions (40 mg.kg^–1^ of 1D9-700DX and irradiation at 4.5 J.cm^–2^) to carefully compare larval survival upon infection with midexponential or early-stationary phase CA- or HA-MRSA inocula, or with inocula of the engineered CA-MRSA strain AH4807 that had been used for the above-described in vitro studies. Indeed, aPDT with 1D9-700DX significantly prolonged larval survival at 24, 48, and 72 hours after irradiation for all 3 MRSA strains grown to midexponential and early-stationary phase (Figure 4, A, C, and E, and Supplemental Figure 7, A, C, and E).

Since a significantly increased survival of *G*. *mellonella* larvae after aPDT was observed, we also examined the bacterial load in the larvae after treatment. Therefore, the numbers of bacteria (CFU/mL) in the larval hemolymph were quantified at 24, 48 and 72 hours after irradiation, using surviving larvae that had, respectively, been infected with the 3 different MRSA strains. As shown in Figure 4, B, D, and F, and Supplemental Figure 7, B, D, and F, the bacterial load was decreased massively at 24 and 48 hours after irradiation, irrespective of the growth stage of the inocula. Of note, an increase in the bacterial load of the treated larvae was observed at 48 hours, which is due to proliferation of residual bacteria that had survived aPDT with 1D9-700DX. At 72 hours after irradiation, the bacterial burden also decreased in untreated surviving larvae, reflecting the clearance of bacteria by the larval innate immune defenses.

### Lack of toxicity of 1D9-700DX toward mammalian cells.

**Figure 5:**
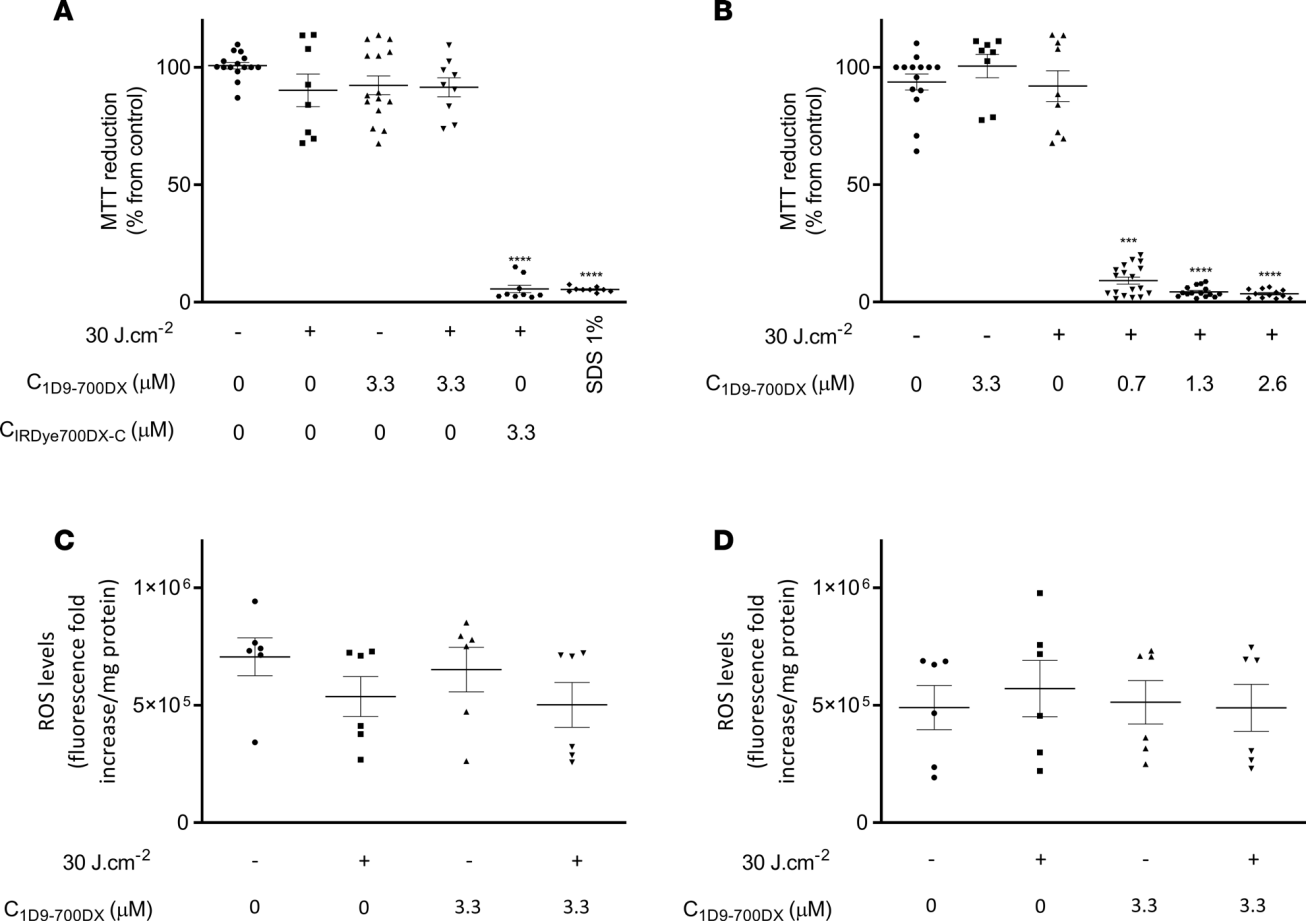
Photo- and cytotoxicity of 1D9-700DX against HeLa cells. (**A**–**D**) HeLa cells were incubated with 1D9-700DX or IRDye700DX carboxylate (IRDye700DX-C) for 30 minutes, and the unbound conjugate was (**A** and **C**) or was not (**B** and **D**) removed by washing with PBS prior treatment with red light (+) at a radiant exposure of 30 J.cm^–2^. (**A** and **B**) Photo- and cytotoxicity was assessed using the colorimetric MTT assay 24 hours after treatment. The percentage of cell viability was calculated relative to viable control cells that were mock-treated with PBS in the dark. Cells treated with 1% were used as a negative control for cell killing. (**C** and **D**) Quantification of H_2_DCFDA fluorescence intensity (*y* axis) by fluorescence spectroscopy immediately after treatment as a measure for ROS production. Data are presented as mean ± SEM of 3 independent experiments performed in triplicates (**A** and **B**) and duplicates (**C** and **D**). Kruskal-Wallis tests with subsequent Dunn′s multiple-comparison tests were used for statistical analysis. Significant differences compared with the control group (no photosensitizer and no light) are marked as follows: ****P < 0.0001.

Since no toxicity of (photoactivated) 1D9-700DX was observed in *G*. *mellonella* larvae, we wanted to evaluate possible cyto- or phototoxic effects of this immunoconjugate toward human cells. To this end, HeLa cells were incubated with 1D9-700DX for 30 minutes; thereafter, the cells were exposed to red light at 30 J.cm^–2^ either directly or after removal of unbound immunoconjugate by washing with PBS. To assess possible cytotoxic effects of 1D9-700DX, control experiments were done where HeLa cells were mock treated with PBS or with 1% SDS and kept in the dark. Cell viability was determined 24 hours after treatment using the colorimetric assay based on the reduction of the yellow MTT by mitochondrial dehydrogenase activity to a purple formazan precipitate. Hence, the reduction of MTT is directly correlated with the metabolic activity of living cells (37). No significant differences in viability were observed for any groups except for the HeLa cells treated with 1D9-700DX (0.7–2.6 μM) and light without the wash, or the SDS-treated HeLa cells (Figure 5, A and B). In particular, exposure of the HeLa cells to red light without removal of the 1D9-700DX resulted in a ~90% reduction of cell viability (Figure 5B), which can be explained by the proximity of the immunoconjugate to the monolayer of the HeLa cells in the 96-well plate. On the contrary, red light treatment after PBS wash showed no significant level of cytotoxicity, even if the 1D9-700DX dose was raised to 3.3 μM. This shows that there was no significant uptake of the 1D9-700DX by the HeLa cells, implying that unwanted side effects of aPDT with this immunoconjugate will be marginal.

As a nontargeted control, the above-mentioned IRDye700DX carboxylate was incubated with the HeLa cells at the same concentration of 3.3 μM that was used for 1D9-700DX. This resulted in ~95% of cell mortality, comparable to 1% SDS control, even after washing with PBS (Figure 5A). This highlights the added value of using targeting agents for the treatment of bacterial infections, as unwanted side effects on mammalian cells can be more readily avoided.

To verify that 1D9-700DX was not opsonized by HeLa cells, we assessed the possible cytosolic production of ROS in HeLa cells treated with this compound, as ROS production would be indicative of the opsonization of 1D9-700DX by the mammalian cells. To this end, the HeLa cells were incubated for 30 minutes with 3.3 μM of 1D9-700DX. Subsequently, PBS-washed or unwashed HeLa cells were incubated with the intracellular ROS-sensitive probe 2’,7’-dichlorohydrofluorescin (H_2_DCFDA), immediately after red light exposure (30 J.cm^–2^). Indeed, no significant ROS production was detectable, neither in the 1D9-700DX– and red light–treated PBS-washed HeLa cells, the unwashed HeLa cells, or the respective negative controls (Figure 5, C and D). This shows that there was no unspecific binding or uptake of 1D9-700DX by the investigated mammalian cells.

### Postmortem aPDT in a human implant model.

**Figure 6:**
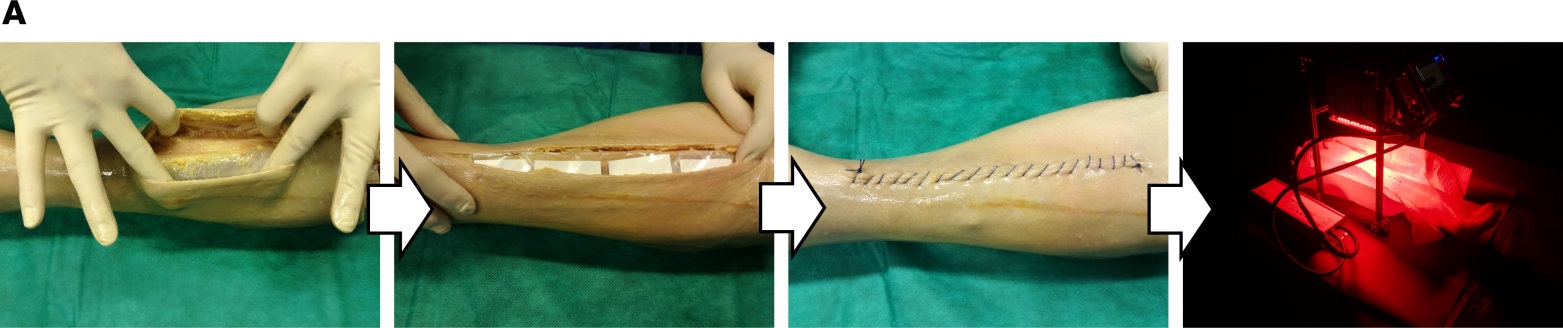
aPDT with 1D9-700DX in a human postmortem infection model. Snap shots of the positioning of nitrocellulose membranes with spotted 1D9-700DX–labeled *S*. *aureus* on the lateral side of the proximal tibia of a human postmortem leg, followed by irradiation with red light at a radiant exposure of 30 J.cm^–2^, or no light exposure. Numbers of surviving bacteria (Log_10_[CFU/mL]) on implanted or nonimplanted nitrocellulose membranes upon treatment with red light at a radiant exposure of 30 J.cm^–2^ (P^+^L^+^) or no light exposure (P^–^L^–^). Overnight-cultures of *S*. *aureus* (clinical isolate) or *S*. *epidermidis* ATCC 35984 (negative control) were incubated with 3.3 μM of 1D9-700DX and spotted on nitrocellulose membranes, which were either kept outside (nonimplanted) or implanted in the human postmortem leg. Bacterial survival was assessed by replica plating of the membranes onto BA plates and overnight incubation at 37°C (*n* = 1 postmortem leg).

The decision to use IRDye700DX as a photosensitizer for the present studies was based on the fact that it absorbs light in the near-infrared range, which has higher penetration in human tissue (~1 cm) than white light (38). To test this property in the context of a human body, a human postmortem model was used that mimics orthopedic implant infections. In our setup, however, we spotted bacteria labeled with 1D9-700DX onto nitrocellulose membranes, which were subsequently implanted subdermally on the proximal tibia of a human cadaver. As a negative control, we spotted a *S*. *epidermidis* strain deficient in 1D9-binding sites on the membranes. Upon skin closure, red light was applied at a radiance exposure of 30 J.cm^–2^ as in the experiments described above (Figure 6). In parallel, duplicate nonimplanted membranes with bacteria labeled with 1D9-700DX were treated with red light at the same radiant exposure. After the irradiation, the implanted and nonimplanted membranes were recollected, and the bacteria were plated for CFU counting. The viable counts of a clinical isolate of *S*. *aureus* after aPDT were substantially reduced from 6.64 to 3.30 log_10_ (CFU/mL). Importantly, placement of the nitrocellulose membrane under the skin and subcutis did not negatively influence the efficiency of the treatment (3.19 log_10_ [CFU/mL] after aPDT). In contrast, no decrease in the CFU counts was observed for *S*. *epidermidis* (6.26 log_10_ [CFU/mL]), as expected, due to its lack of 1D9-binding sites. Together, these observations provide proof-of-principle that 1D9-700DX is applicable for use in the treatment of subdermal *S*. *aureus* infections in humans.

### Full eradication of MRSA in human plasma by aPDT with 1D9-700DX.

**Figure 7:**
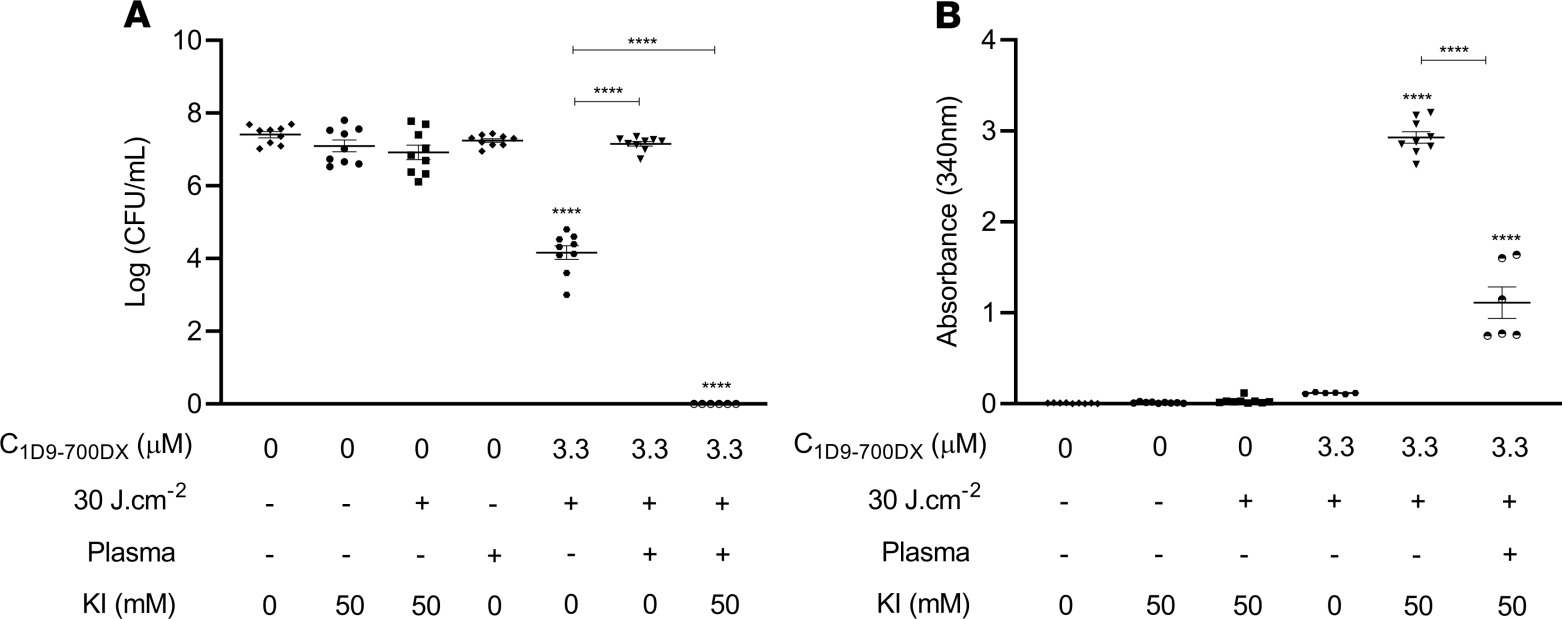
aPDT with 1D9-700DX in combination with KI in the presence of plasma. Photoactivated killing of CA-MRSA AH4807 grown to exponential phase (~1 × 10^7^ CFU/mL), in the presence or absence of plasma, upon treatment with or without 3.3 μM of 1D9-700DX and with or without 50 mM of KI. Bacteria were treated with red light at a radiant exposure of 30 J.cm^–2^ (+) or kept in the dark (–). (**A** and **B**) Numbers of surviving bacteria (Log_10_[CFU/mL]) and monitoring of the formation of iodine at 340 nm are represented in **A** and **B**, respectively. Data are presented as mean ± SEM of 3 experiments performed in triplicate. Ordinary 1-way ANOVA tests with subsequent Dunnett’s multiple-comparison tests were used for statistical analysis. Significant differences compared with the negative control group (no 1D9-700DX and no light) are marked as follows: ****P < 0.0001.

The human body is very well protected against the detrimental effects of ROS, with a major contribution to extracellular antioxidant activity by HSA. In particular, the free thiol residues present in HSA are known to trap multiple ROS (39), which could compromise the aPDT efficacy of 1D9-700DX in the treatment of *S*. *aureus* infections. Therefore, the antioxidant effect of human plasma was investigated ex vivo by treating the CA-MRSA strain AH4807 with 3.3 μM of 1D9-700DX and red light at 30 J.cm^–2^ upon suspension in plasma, a dose that kills 50% of 2 × 10^7^ CFU/mL of the bacteria in PBS. Consistent with its presumed antioxidant activity, the plasma fully blocked the aPDT effect of 1D9-700DX (Figure 7A). Importantly, however, it is known that aPDT can be potentiated with KI due to the reaction of this compound with ^1^O_2_, which leads to the formation of peroxyiodide that is subsequently decomposed into free iodine and H_2_O_2_ (40). Indeed, as shown in Figure 7A, 3.3 μM of 1D9-700DX combined with 50 mM KI eradicated 100% of the bacteria in PBS after exposure to red light, representing a 4-log enhanced efficacy compared with 1D9-700DX alone. In the presence of plasma, the effect of KI was even more drastic, with an approximately 8-log enhanced aPDT efficacy that led to complete eradication of the bacterial inoculum (Figure 7A). To verify the proposed mode of action of KI, the formation of iodine upon aPDT was quantified by measuring the absorbance at 340 nm. This showed that the presence of plasma decreased the iodine formation to half (Figure 7B). Nonetheless, this was sufficient to achieved complete killing of the CA-MRSA AH4807 inoculum.

## Discussion

The present study was motivated by the pressing need to develop antimicrobial agents with alternative modes of action to treat persistent *S*. *aureus* infections due to acquired antibiotic resistance and biofilm formation. In this context, bacteria-targeted aPDT could become a valuable complement to the currently applied antibiotic therapies, especially since bacterial resistance to potent photosensitizers has not yet been reported. To produce a specific targeted aPDT agent, we conjugated a human IgG1 (1D9) with high specificity for *S*. *aureus* (30) to a near-infrared photosensitizer (IRDye700DX) that is currently applied in cancer therapy (23–26). This immunoconjugate (1D9-700DX) demonstrated to be photostable upon irradiation with red light and a high producer of ^1^O_2_ upon photoactivation. Importantly, 1D9-700DX was shown to be active at the *S*. *aureus* cell wall, allowing the complete eradication of clinical isolates at 1 × 10^7^ CFU/mL, a dose that was previously proven to be highly infective (41). In addition, 1D9-700DX effectively killed all tested *S*. *aureus* strains, including MRSA, irrespective of variations in the expression level of the 1D9 target protein IsaA, which is strictly conserved and invariably presented on the surface of all clinical *S*. *aureus* isolates investigated to date (14–18).

Our present observations show that targeted aPDT with 1D9-700DX is very effective in destroying the outer layers of *S*. *aureus* biofilms to aid penetration. This is an important finding because biofilms are multilayered structures that protect the embedded bacteria against the host immune system and antimicrobial agents. It thus seems that aPDT with 1D9-700DX could be a beneficial approach to “crack” staphylococcal biofilms, which would thus become easier targets for the host immune system and antibiotic therapy.

*G. mellonella* insect larvae have been increasingly used as a rapid model to screen potentially novel antimicrobial drug candidates (34). In the present study, aPDT with 1D9-700DX was shown to be highly effective in treating MRSA-infected *G*. *mellonella* larvae. Although we observed a relapse in the bacterial burden 48 hours after aPDT, this relapse was not lethal to the larvae, as evidenced by the increased survival rates (~80%) at 72 hours after treatment. The latter must be attributed to the larval innate immune defenses. Together, these findings show that, at least in the larvae of *G*. *mellonella*, aPDT with 1D9-700DX reduces the bacterial burden to such an extent that the host’s immune responses can overcome infections caused by multidrug resistant *S*. *aureus*. Of note, the *G*. *mellonella* infection model adheres to the principles of replacement, reduction, and refinement (known as 3Rs) and can potentially reduce the number of vertebrates used for experimental infection studies.

To enable the future clinical application of 1D9-700DX aPDT, possible photo- and cytotoxic effects must be verified. The immunoconjugate displayed no cytotoxicity against HeLa cells in the absence of activation with red light. Nonetheless, when 1D9-700DX was photoactivated, it did affect the viability of HeLa cells if the unbound immunoconjugate was not removed, since the production of ROS in the proximity of tumor cells causes their killing. Notably, however, the HeLa cell viability was not affected when unbound 1D9-700DX was washed away with PBS, which mimics the in vivo situation where the blood circulation will “wash” away any unbound immunoconjugate from a site of infection, with the exception of the causative bacterial agent that is targeted by the immunoconjugate. Importantly, the nontargeted IRDye700DX carboxylate strongly affected HeLa cell viability, despite a washing step with PBS. This argues in favor of using targeted agents for aPDT of bacterial infections, as this will minimize adverse effects. In addition, our present findings show that 1D9-700DX is not easily taken up by mammalian cells and, therefore, does not cause intracellular ROS production. Instead, this immunoconjugate is highly specific for IsaA-expressing bacteria.

Penetration of light into human tissue is one of the main hurdles for aPDT. Compared with classical photosensitizers, near-infrared photosensitizers allow a deeper light penetration. Accordingly, the present postmortem experiments show that human skin does not pose a barrier to photoactivation of 1D9-700DX and the consequent subdermal destruction of *S*. *aureus*. Of note, the postmortem set-up lacks blood circulation, and the overall conditions for aPDT are therefore different from the actual clinical setting. Furthermore, it is important to bear in mind that the maximum penetration depth for red light is about 1 cm (38, 41). This implies that aPDT of deeper-seated infections with 1D9-700DX would require either red light delivery through an endoscopic device or an intraoperative approach. In the latter scenario, the near-infrared fluorescence of the 1D9-700DX photosensitizer would allow image-guided surgical debridement of infected tissues or implants, as recently showcased for 1D9 labeled with the fluorophore IRDye800CW (20). Of note, while IRDye800CW is a potent fluorophore, it is not a suitable agent for aPDT, unlike IRDye700DX, as demonstrated in the present study.

Last, the human body is very well protected against oxidative damage. This is exemplified by the antioxidant properties of HSA, which is the most abundant protein in human plasma. Upon secretion into the bloodstream, it presents sulfur-containing methionine and cysteine residues, which are responsible for more than 70% of the free radical–trapping activity of serum (39). Methionine is particularly susceptible to oxidation, which leads to the production of methionine sulfoxide, representing an endogenous antioxidant defense mechanism for proteins. Even more so, the cysteine residue 34 of HSA constitutes the large pool of free thiols in the human body. Although the scavenging of ROS is of critical importance for human health, the strong antioxidant activity as presented by HSA is a potential hurdle for aPDT with 1D9-700DX in clinical settings. This applies in particular to aPDT in the context of image-guided debridement of infected implants and tissue, where blood will be present due to the surgical procedure (20, 21). Indeed, we show that the presence of human plasma with its high HSA content can seriously interfere with aPDT using 1D9-700DX. However, this antioxidant activity can be completely overcome through the application of KI, a nontoxic salt that was previously shown to improve the aPDT efficacy in several models (42–45). Importantly, KI has been approved by the US Food and Drug Administration for thyroid blocking in radiation emergencies, which underpins its therapeutic application potential.

Altogether, we have shown that aPDT with 1D9-700DX can target and destroy highly drug-resistant *S*. *aureus* strains, irrespective of their presence in a planktonic state or a biofilm community. We furthermore show in a human postmortem implant infection model that aPDT with 1D9-700DX can be achieved subdermally. Moreover, the efficacy in human plasma can be enhanced with KI to such an extent that the intrinsic defenses against oxidative damage are overcome and MRSA inocula of more than 1 × 10^7^ CFU/mL are fully eradicated. We therefore believe that 1D9-700DX holds great promise for clinical application in all infection scenarios where *S*. *aureus* can be exposed to red light.

## Methods

Supplemental Methods and complete unedited blots are available online with this article.

### Binding of 1D9 to S. aureus.

*S*. *aureus* WT strains SH1000 and Newman, and the respective isogenic mutant strains MS001 (Δ*isaA*) and Newman Δ*spA*, Δ*sbi* (16), were grown overnight in tryptic soy broth (TSB; Oxoid) in a shaking incubator at 37°C. Overnight cultures were diluted to an OD_600_ of 1. Cells were collected by centrifugation at 16,000*g* for 2 minutes at room temperature and washed with PBS. The Alexa Fluor 555 dye (Thermo Fisher Scientific) was cross-linked to the human mAb 1D9 via activated NHS ester chemistry. Cells were incubated with 3 μg.mL^–1^ of the 1D9-Alexa555 immunoconjugate for 30 minutes in PBS. To remove unbound antibody, the cells were washed with PBS and, thereafter, fixed in 4% paraformaldehyde in PBS for 10 minutes. Last, the bacterial DNA was stained with DAPI (Roche) followed by 1 PBS wash. Cells were spotted on a glass slide for microscopy. Image acquisition was performed with a Leica confocal laser scanning microscope (DMI 6000, SP8). The recorded images were processed using ImageJ software (NIH). Intensity surface plots were created using FIRE LUT and the surface plot tool in ImageJ.

### Visualization of S. aureus cell surface targeting by 1D9-700DX through TEM.

The WT *S*. *aureus* strain Newman was cultured overnight in TSB at 37°C. A fresh subculture was grown to exponential phase (OD = ~0.7) and then harvested and washed with PBS by centrifuging for 2 minutes at 16,000*g* at room temperature. The bacterial pellet was resuspended with 1D9-700DX (1.3 μM) and incubated for 30 minutes at room temperature (RT). After incubation with the drug, 1 mg.mL^–1^ of DAB in sodium cacodylate buffer, pH 7.4, was added, and the bacterial suspension was irradiated with red light at a radiant exposure of 60 J.cm^–2^ (10 minutes irradiation, 100 mW.cm^–2^). A control sample consisting of bacteria exposed to light and DAB only (P^–^L^+^ DAB^+^) was included. The sample preparation for TEM was performed as described previously (46). Briefly, the samples were fixed in 2% glutaraldehyde with 0.5% paraformaldehyde in 0.1M of cacodylate buffer, pH 7.4, overnight, at 4°C, followed by washing in 0.1M of cacodylate buffer. Then, the samples were postfixed with 1% osmium tetroxide/potassium ferrocyanide for 2 hours, at 4°C, and dehydrated in a graded series of ethanol. After embedding in Epon resin (Serva), thin sections (60 nm) were cut with an ultramicrotome UC7 (Leica) and collected on 150 mesh copper grids. Sections were not contrasted with uranyl or lead. Images were recorded with a FEI Cm100 TEM operated at 80 KV using a Morada digital camera. The recorded images were processed using ImageJ software.

### Phototoxicity assay on planktonic bacteria.

Two bioluminescent *S*. *aureus* strains (Xen36 and AH4807) were used in this study together with the laboratory strains SH1000 and MS001 (Supplemental Table 1). The MSSA strain Xen36 was previously derived from the parental *S*. *aureus* strain ATCC-49525, a clinical isolate from a septic patient, in which the *lux* operon from *Photorhabdus luminescens* was modified for Gram-positive bacterial expression and integrated into the host’s native plasmid (47). The MRSA strain AH4807 was derived from the CA-MRSA LAC strain AH126353, where the *P*. *luminescens lux* operon was again modified for Gram-positive bacterial expression and integrated at the φ11 attachment site on a plasmid of the host (48). *S*. *aureus* strains were grown to exponential phase (OD_600_ = 0.5) and then harvested and washed with PBS by centrifugation for 2 minutes at 16,000*g* at room temperature. The bacteria were 10-fold diluted, and 50 μL aliquots (~2 × 10^7^ CFU/mL) were incubated with different concentrations of 1D9-700DX (0.7–9.8 μM) or PBS in a 96-well plate at RT for 30 minutes in the dark, and then kept in the dark or exposed to 30 J.cm^–2^ of red light. For the time-response study, bacteria were incubated with 4.9 μM of 1D9-700DX and subjected to red light at a radiant exposure that ranged between 0 and 30 J.cm^–2^. After treatment, bacteria were serially diluted in PBS, plated on blood-agar (BA) plates, and then incubated aerobically for 16 hours at 37°C for CFU counting. Visualization of bioluminescence and fluorescence with the IVIS Lumina II (PerkinElmer) was additionally performed with a higher bacterial burden (~1 × 10^8^ CFU/mL) to allow proper visualization.

### H_2_O_2_ production after aPDT.

*S*. *aureus* Xen36 and AH4807, grown as described above, were incubated with 6 μM of 1D9-700DX and subjected to red light at a radiant exposure of 30 J.cm^–2^. H_2_O_2_ was detected with 10 μM of an AquaSpark Peroxide Probe (Biosynth Carbosynth), which is a dioxetane-based substrate for luminescence detection of H_2_O_2_ and other peroxides in biological samples. Visualization of bioluminescence was performed with the IVIS Lumina II, and the total flux (photons/second) was determined by region of interest quantification with the Living Image 4.5.5 software (PerkinElmer).

### Biofilm targeting by aPDT.

For biofilm formation, a *S*. *aureus* NCTC 8325-4 WT overnight culture was diluted 1:50 in TSB supplemented with 5% glucose and 4% sodium chloride, and 500 μL aliquots were used to inoculate 24-well plates containing 13 mm chemically resistant borosilicate glass coverslips. Upon 24-hour incubation at 37°C, planktonic bacteria on top of the coverslips were washed away with PBS. The biofilms were then incubated with 1D9-700DX (15.5 μM) for 30 minutes at RT. After washing with PBS to remove the unbound immunoconjugate, biofilms were treated with red light at a radiant exposure of 30 J.cm^–2^ and stained with the LIVE/DEAD BacLight Bacterial Viability Kit (Thermo Fisher Scientific). Briefly, this kit contains 2 dyes: the Syto9 dye penetrates both viable and nonviable bacteria, while the propidium iodide penetrates bacteria with damaged membranes and quenches the fluorescence of Syto9. LIVE/DEAD-stained biofilms were examined using a Leica confocal laser scanning microscope (DMI 6000, SP8). The recorded images were processed using ImageJ software.

### In vivo G. mellonella survival assay after aPDT.

Bacterial cultivation and infection of *G*. *mellonella* larvae were performed as detailed in the Supplemental Materials. Ninety minutes after infection, 10 μL of 40 mg.kg^–1^ of 1D9-700DX or PBS were administered to the larvae, and after 30 minutes of incubation, the aPDT group (P^+^L^+^) was treated with red light at a radiant exposure of 4.5 J.cm^–2^. Viability was scored according to the *G*. *mellonella* Health Index Scoring System (49) at 0, 24, 48, and 72 hours after treatment based on pigmentation and mobility.

### Persistence of S. aureus in G. mellonella hemolymph after aPDT.

The number of bacterial cells in the *G*. *mellonella* hemolymph was determined as described previously (50). Briefly, at 0, 24, 48, and 72 hours after aPDT treatment, 3 surviving larvae per group were bled with a scalpel and squeezed to remove the hemolymph in a final volume of approximately 100 μL. The hemolymph was homogenized and centrifuged at 2,513*g* for 10 minutes at room temperature. Serial dilutions were plated on tryptic-soy agar (TSA) plates for CFU counting. All experiments were repeated at least twice, and representative experiments are shown.

### Cytotoxicity and phototoxicity of 1D9-700DX in mammalian cells.

The human cervical cancer HeLa cell line (ATCC) was cultured in DMEM-GlutaMAX medium (Thermo Fisher Scientific) supplemented with 10% of FBS, at 37°C and 5% CO_2_. A total of 0.25% Trypsin-EDTA (Thermo Fisher Scientific) was used to detach adherent cells for subculturing. Cells were seeded into 96-well cell culture plates at a density of 3 × 10^4^ cells/well. On the following day, cells were treated with 1D9-700DX or IRDye700DX carboxylate in the dark, for 30 minutes. One group was washed with PBS, and fresh DMEM medium was added, while the other group remained with the drug in PBS. Irradiation with 30 J.cm^–2^ of red light was subsequently performed. Cell metabolic activity at 24 hours after aPDT was determined with the MTT assay, which measures the ability of HeLa cells to reduce MTT (MilliporeSigma) to colored formazan crystals. The formation of formazan was quantified with a microplate spectrophotometer (Synergy HT, Biotek instruments) by measuring the absorbance at 570 nm, using 620 nm as the background wavelength. The percentage of absorbance for each treated sample was normalized to each untreated control.

### Detection of intracellular ROS generation in mammalian cells after aPDT.

After aPDT, HeLa cells were incubated with 5 μM of H_2_DCFDA (Invitrogen), a chemically reduced form of fluorescein used as an indicator for intracellular ROS generation for 1 hour at 37°C in the dark. After incubation with H_2_DCFDA, cells were washed with PBS and mechanically scraped and resuspended in 1% (m/v) SDS solution in PBS. Subsequently, the fluorescence was measured using a Synergy HT microtiter plate reader with excitation and emission filters of 485/20 nm and 540/25 nm. Protein concentrations were determined using the Pierce BCA Protein Assay Kit.

### Human postmortem implant model.

Dilution series of overnight cultures of an *S*. *aureus* clinical isolate or *S*. *epidermidis* ATCC 38984 were treated with 3.3 μM of 1D9-700DX and spotted on nitrocellulose membranes. The membranes were subsequently implanted on the lateral side of the proximal tibia of a human postmortem leg, which was subsequently irradiated with 30 J.cm^–2^ of red light. Nonimplanted control membranes were either irradiated or kept in the dark. Subsequently, the membranes were spotted onto BA plates for CFU counting.

### Combination therapy of aPDT with 1D9-700DX and KI in the presence of plasma.

CA-MRSA AH4807 was grown to exponential phase (OD_600_ = 0.5), and they were then harvested and washed with PBS by centrifugation for 2 minutes at 16,000*g* at room temperature. The bacteria were 10-fold diluted and 50 μL aliquots (~2 × 10^7^ CFU/mL) were incubated with 50 μL of a mixture of 3.3 μM of 1D9-700DX or PBS and/or 50 mM of KI (MilliporeSigma) or PBS and/or plasma in a 96-well plate at RT for 30 minutes in the dark. The mixtures with bacteria were then either kept in the dark or exposed to 30 J.cm^–2^ of red light. After light exposure, the bacteria were serially diluted in PBS, plated on BA plates, and then incubated aerobically for 16 hours at 37°C for CFU counting. The generation of iodine after irradiation was monitored by reading the absorbance of 96-well plates at 340 nm with a microplate spectrophotometer (Synergy HT).

### Statistics.

The results are presented as mean ± SEM. All statistical analyses were performed with GraphPad Prism 8.0.1. Statistical significance among 2 unpaired groups was performed with the Mann-Whitney *U* test. Assessing a variable in 3 or more unmatched groups was performed with Kruskal-Wallis tests in the case of cytotoxicity studies with mammalian cells, or with ordinary 1-way ANOVA tests in case of combination approaches with 1D9-700DX and KI in the presence of plasma. Subsequently, the Dunn’s or the Dunnett’s multiple-comparison tests were performed. Two conditions (bacterial viability studies) were assessed using 2-way ANOVA tests and subsequently by the Šidák multiple-comparison test. The statistical significance of differences in the killing of *G*. *mellonella* larvae by *S*. *aureus* was assessed by Gehan-Breslow-Wilcoxon test. *P* < 0.05 was considered significant.

### Study approval.

All postmortem experiments were conducted according to institutional guidelines with prior approval from the scientific review committee of the Skills Center of the University Medical Center Groningen, the Netherlands, and according to the applicable law (“Wet op de Lijkbezorging”, Art 18, lid 1 and 19, BWBR0005009). All individuals involved in the human postmortem studies have provided informed written consent for the use of their bodies for scientific research and teaching. Blood donations from healthy volunteers were collected with approval of the medical ethics committee of the UMCG (approval no. METc 2016/621) and after written informed consent, in accordance with the Declaration of Helsinki guidelines and local regulations.

## Author contributions

MB and JMVD conceived and designed the experiments. MB, AAS, SS, EJMR, MLA, MH, and WS performed experiments and analyzed the data. FRP, ARH, and KPF contributed strains and reagents. GB, GMVD, MVO, and JMVD supervised the project. MB and JMVD wrote the manuscript. All authors have read and approved the manuscript.

## Supplementary Material

supplemental data

supplemental Video 1

## Version 1 10/13/2020

In-Press Preview

## Version 2 11/19/2020

Electronic publication
