# Supplementary material for: Fighting Staphylococcus aureus infections with light and photoimmunoconjugates
Source: JCI Insight. 2020 Nov 19;5(22):e139512. doi: 10.1172/jci.insight.139512 (PMC7710284; doi:10.1172/jci.insight.139512)
Supplement: supplemental data [file jciinsight-5-139512-s111.pdf]

Supplementary Information

Fighting *Staphylococcus aureus* infections with light and photo-immunoconjugates

Mafalda Bispo[1], Andrea Anaya-Sanchez[1], Sabrina Suhani[1], Elisa J. M. Raineri[1], Marina López-Álvarez[1], Marjolein Heuker[1], Wiktor Szymański[2,3], Francisco Romero Pastrana[1], Girbe Buist[1], Alexander R. Horswill[4], Kevin P. Francis[5], Gooitzen M. van Dam[6], Marleen van Oosten[1], Jan Maarten van Dijl[1]*

[1]Department of Medical Microbiology, University of Groningen, University Medical Center Groningen, Groningen, The Netherlands

[2]Department of Radiology, Medical Imaging Center, University of Groningen, University Medical Center Groningen, Groningen, The Netherlands

[3]Stratingh Institute for Chemistry, University of Groningen, Groningen, The Netherlands

[4]Department of Immunology and Microbiology, University of Colorado Anschutz Medical Campus, Aurora, Colorado, USA.

[5]PerkinElmer, Hopkinton, Massachusetts, USA

[6]Department of Surgery, Division of Surgical Oncology, Nuclear Medicine and Molecular Imaging, Intensive Care, University of Groningen, University Medical Center Groningen, Groningen, The Netherlands

**Address correspondence to Jan Maarten van Dijl, Department of Medical Microbiology, University Medical Center Groningen, Hanzeplein 1, 9700 RB Groningen, The Netherlands, Phone: +31503615187, E-mail: j.m.van.dijl01@umcg.nl*

Supplementary Methods

1D9 monoclonal antibody conjugation with IRDye700DX

The human mAb 1D9 was produced as previously described (1) by transient transfection of Expi293F cells (Life Technologies). IRDye® 700DX (LiCor Biosciences) was cross-linked to the human mAb 1D9 via activated *N*-hydroxysuccinimide (NHS) ester chemistry according to the manufacturer's instructions. Lithium dodecyl sulfate (LDS)-PAGE analysis of the 1D9-700DX immunoconjugate was performed using a 10% Bis-Tris gel (NuPAGE gels, Life Technologies) followed by Simply Blue staining (Simply Blue Safe Stain, Life Technologies), or fluorescence imaging with an Amersham™ Typhoon™ Biomolecular Imager (GE Healthcare). A reducing agent (NuPAGE, ThermoFisher) was employed to separate the heavy and light chains, and to improve the band resolution.

Enzyme-linked immunosorbent assay (ELISA)

Immunoreactivity of the 1D9-700DX compared to the native mAb 1D9 was assessed using an ELISA. To this end, a 96-well plate with high binding capacity (Greiner Bio-one) was coated with 10 $ng.ml^{-1}$ of purified immunodominant antigen A (IsaA) (2) at room temperature (RT), overnight. After three washing steps with PBS/T (pH 7.4), unspecific binding sites were blocked with 0.1% BSA (Sigma-Aldrich) diluted in PBS (pH 7.4). The native antibody or the immunoconjugate were applied to each well at a concentration of 2 $\mu g.mL^{-1}$, followed by incubation for 2 hours. After three washes, a goat anti-human IgG labelled with horse radish peroxidase (HRP) was added. After washing, HRP was detected by adding 100 µL of 3,3',5,5'-tetramethybenzidine (TMB/E, Merck) solution for 30 min. The reaction was stopped by adding 50 µL of 1 M sulphuric acid (H_2SO_4). Absorbance was measured using a microplate reader at 450 nm (Synergy™ HT, Biotek instruments).

Photostability of 1D9-700DX

A solution of 9.8 µM of 1D9-700DX was prepared in PBS and its photostability was determined by monitoring the decrease of the Q band absorbance between 550 and 750 nm, upon irradiation for different periods of time (0-30 min) using a Standardized Light Emitting Diode device with a 690-nm high-output (n=126) (3). The irradiance was 100 $mW.cm^{-2}$.

Generation of singlet oxygen by 1D9-700DX

The production of singlet oxygen was assessed by measuring the absorbance decay of 1,3-diphenylisobenzofuran (DPBF, Sigma-Aldrich) due to $^1O_2$ scavenging. Solutions containing DPBF (200 µM) with or without IRDye700DX or 1D9-700DX (2 µM) were prepared in DMSO and then irradiated at RT with red light at 10 $mW.cm^{-2}$. DPBF absorbance was recorded at 415 nm. The results were expressed by plotting the DPBF depletion against the irradiation time.

Western blotting

Bacterial overnight cultures of *S. aureus* Xen36, AH4807, SH1000 and MS001 were diluted to an OD_{600} of 2 and harvested by centrifugation at 16,000 rpm for 2 min at 4°C. The bacterial pellet was washed with PBS, resuspended in LDS sample buffer (Life Technologies), and the cells were disrupted with 0.1 mm glass beads (Biospec Products) in a Precellys 24 homogenizer

(Bertin Technologies). Thereafter, proteins were denatured at 95°C for 10 min. Then, samples were analyzed by LDS-PAGE and proteins were visualized by Simply Blue protein staining, or by blotting onto Protan nitrocellulose transfer paper (Whatman) and subsequent immunodetection with IRDye800CW-labeled 1D9 antibody. The fluorescence of the blots was visualized with an Amersham™ Typhoon™ Biomolecular Imager using the 800 nm channel. Fluorescence quantification of the detected protein bands was performed with Image J software.

Production of IRDye700DX carboxylate

IRDye® 700DX NHS ester (LiCor Biosciences) was incubated with Milli-Q water for one week at RT, and its hydrolysis was confirmed by high performance liquid chromatography (HPLC) analysis with photodiode-array (PDA) detection. HPLC analyses were performed on Shimadzu equipment. The eluents were acetonitrile and water with triethylammonium acetate (10 mM). For reaction monitoring, a XTerra MS C18 column (3.5 μm, 125 Å, 3.0 x 150 mm) in combination with a PDA detector (190 nm – 800 nm) was used with an elution gradient from 5% to 80% organic phase.

Binding affinity and phototoxicity assay on planktonic bacteria

S. aureus AH4807 (MRSA) was grown to exponential phase (OD_{600}=0.5), and then harvested and washed with PBS by centrifugation for 2 min at 16,000 x g. The bacteria were incubated with 9.8 μM of 1D9-700DX, IRDye700DX carboxylate or PBS, at RT for 30 min in the dark. The bacteria were washed once with PBS to remove unbound compounds, and then plated in a 96-well plate for fluorescence imaging in the Typhoon™ Biomolecular Imager. Next, bacteria were either kept in the dark or exposed to 30 $J.cm^{-2}$ (5 min irradiation, 100 $mW.cm^{-2}$) of red light. After treatment, bacteria were serially diluted in PBS, plated on blood-agar (BA) plates, and then incubated aerobically for 16 h at 37°C for counting of colony-forming units (CFU).

Virulence assessment of *S. aureus* strains and aPDT efficacy in a *Galleria mellonella* infection model

Bacterial cultivation and infection of *Galleria mellonella* larvae were performed as previously described (4). Briefly, *S. aureus* USA300 D15 (CA-MRSA), *S. aureus* USA300 D17 (HA-MRSA) expressing GFP (10 $\mu g.mL^{-1}$ chloramphenicol), kindly provided by Solomon Mekonnen and Uwe Völker (University of Greifswald, Greifswald, Germany) or *S. aureus* CA-MRSA AH4807 were grown overnight in TSB at 37°C. Pre-cultures and main cultures were prepared the following day in 10 mL of RPMI medium supplemented with 2 mM glutamine (GE Healthcare/PAA). When the cultures reached the mid-exponential growth phase (OD_{600} of ~0.5) or the early-stationary growth phase (OD_{600} of ~1.1), the bacteria were harvested by centrifugation at 7000 rpm for 20 min at 4°C. The pelleted cells were washed in PBS, collected by centrifugation, resuspended in PBS, and diluted to the desired number of CFU per mL. Different doses of bacteria were injected in the *G. mellonella* larvae (Frits Kuiper Groningen) at a volume of 10 μL using an insulin pen (NovoPen 5 of Novo Nordisk™). Subsequently, the larvae were incubated at 37°C to assess each strain's virulence at mid-exponential or early-stationary growth phase (10^5, 10^6 and 10^7 CFU/larva). For each group, 10 larvae were used, and two control groups were included for each experiment: one injected with PBS as a control for physical trauma and another without injection as a control for general viability.

For the aPDT studies, suspensions of bacteria harvested in the mid-exponential growth phase (10^6 CFU/larva), or the early stationary growing phase (10^5 CFU/larva) were injected into the last pro-leg of the larvae. For each experimental condition, a group of 10 larvae was used. The ideal 1D9-700DX concentration and light dose were determined by dose-responses studies in infected and uninfected larvae, respectively.

Supplementary Figures and Tables

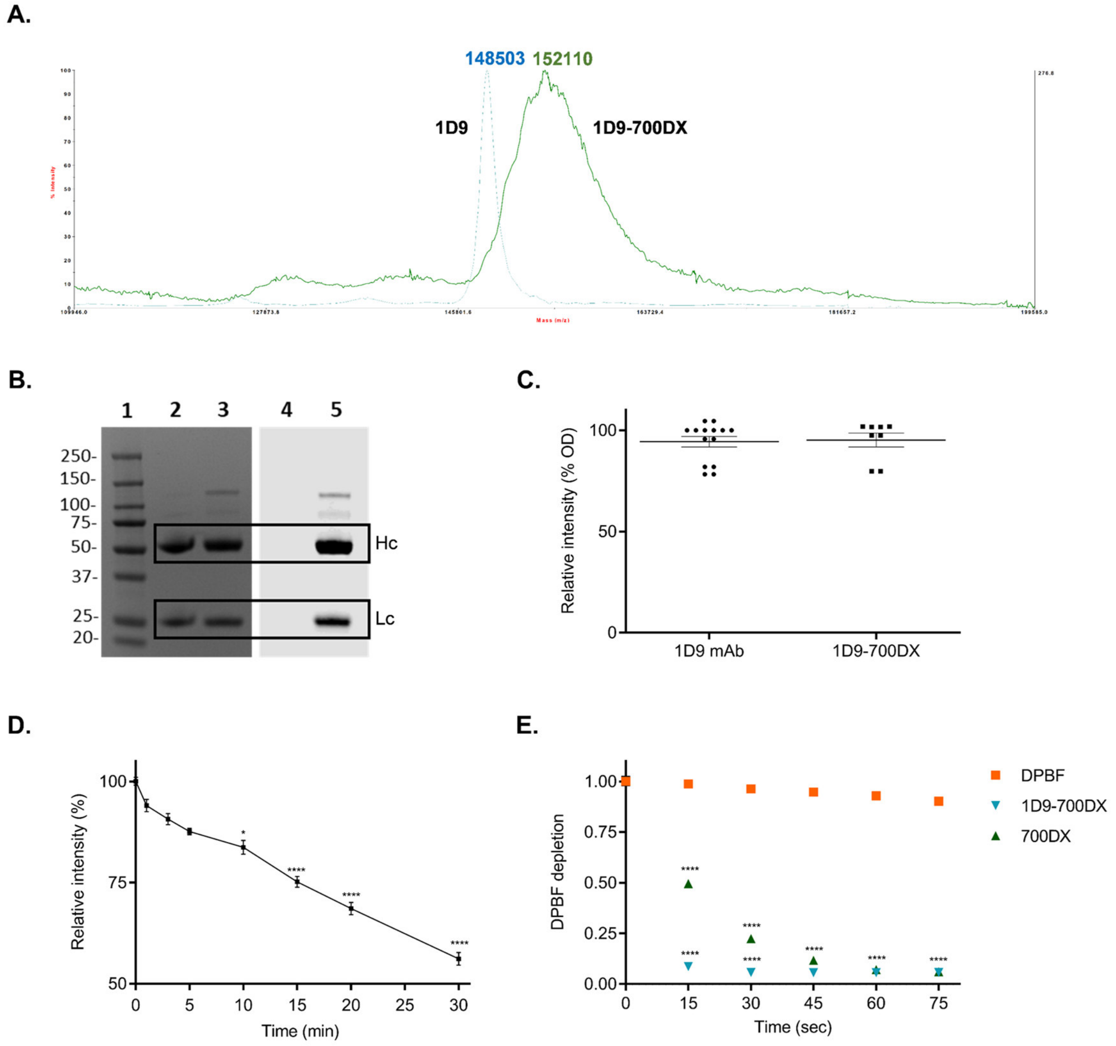

Figure S1. **Characterization of the 1D9-700DX immunoconjugate.** (**A**) MALDI-TOF spectra of the monoclonal antibody 1D9 and the immunoconjugate 1D9-700DX. (**B**) LDS-PAGE analysis of 1D9-700DX in a reducing 10% Bis-Tris gel. The heavy (Hc) and light chains (Lc) in lanes 2 and 3 were visualized by Simply Blue staining (left gel) and fluorescent bands in lane 5 were visualized by scanning with an Amersham™ Typhoon™ Biomolecular Imager (right gel). Lane 1: Molecular weight ladder (MW in kDa); lanes 2 and 4: reduced native 1D9 antibody; lanes 3 and 5: reduced 1D9-700DX. (**C**) Comparative ELISA of the native 1D9 monoclonal antibody and 1D9-700DX. Data is represented as relative intensity of the absorbance at 450 nm of 1D9 native antibody and of 1D9-700DX. (**D**) Photostability of IRDye700DX (9.8 µM) in PBS after exposure to red light at an irradiance of 100 mW.cm^{-2} for different periods of time (0-30 min). The results are presented as a percentage of the ratio of residual absorbance at 689 nm after and prior irradiation for different periods of time. (**E**) Photo-oxidation of DPBF (200 µM) in DMSO with or without IRDye700DX or 1D9-700DX (2 µM) upon irradiation for different periods of time with a high-output LED (690 nm, 10 mW.cm^{-2}). DPBF absorbance was recorded at 415 nm. Data are the mean value ± SEM of three independent experiments performed in triplicates (**C-E**). Two-way ANOVA tests with subsequent Turkey's multiple comparisons tests were used for statistical analysis of the DBPF photo-oxidation. Ordinary one-way ANOVA tests with subsequent Dunnett's multiple comparisons tests were used for statistical analysis of IRDye700DX photostability. Significant differences compared to t = 0 min (**D**) or DPBF (**E**) are marked as follows: *, p<0.03; **, p<0.002; ***, p<0.0002; ****, p < 0.0001.

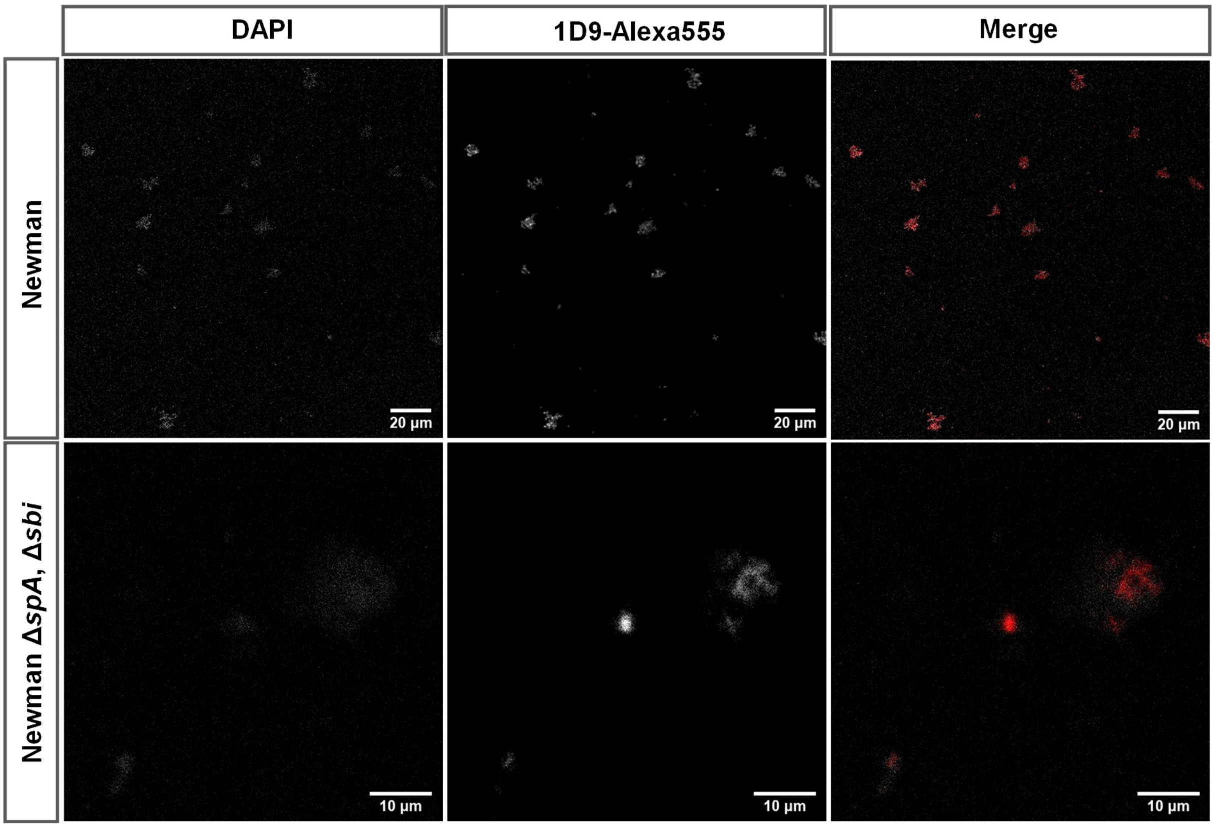

Figure S2. IsaA-specific targeting of 1D9-based immunoconjugates to the *S. aureus* cell surface. Co-localization of the 1D9-Alexa555 (red) immunoconjugate with *S. aureus* Newman wild-type and Newman Δ*spA*, Δ*sbi* stained with DAPI (grey). 3 µg.mL^{-1} of 1D9-Alexa555 were incubated with diluted bacterial overnight cultures (OD_{600} = 1) and imaged with a confocal laser scanning microscope.

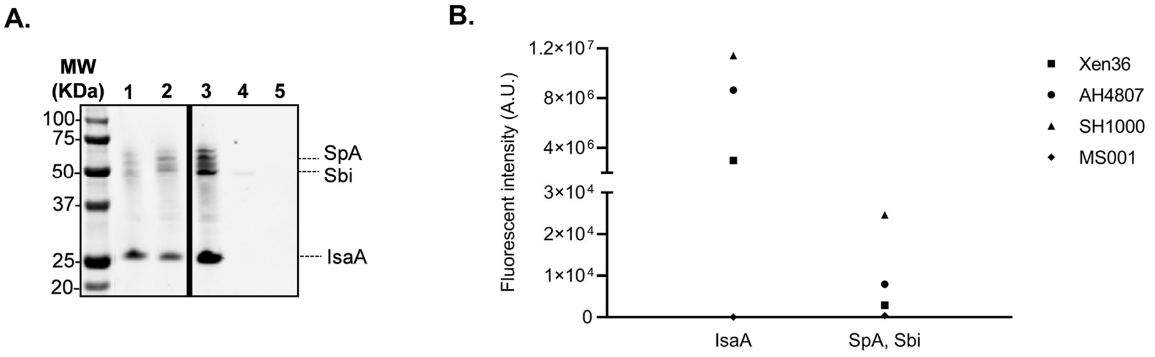

Figure S3. **Western blotting analysis to verify 1D9 binding by different *S. aureus* strains and susceptibility of *S. aureus* SH1000 to aPDT.** (**A**) Western blotting and subsequent immunodetection of 1D9-800CW binding to the cell-associated forms of *S. aureus* IsaA and the IgG-binding proteins SpA and Sbi using different strains: lane 1, *S. aureus* Xen36; lane 2, AH4807 [MRSA]; lane 3, SH1000; lane 4, MS001 Δ*isaA*; lane 5, *E. coli* ATCC 25922. Of note, lanes 2 and 3 were run on the same gel but were noncontiguous. (**B**) Fluorescence quantification of 1D9-800CW binding to IsaA, SpA and Sbi on the Western blot presented in (**A**).

A.

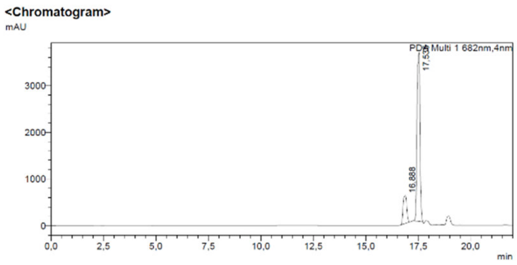

<Peak Table>

PDA Ch1 682nm

Peak#	Ret. Time	Area	Height	Conc.	Unit	Mark	Name
1	16,888	7150939	597540	0,000	mg/L	M	IRDye700DX-COOH
2	17,535	35006963	3615651	0,000	mg/L	M	IRDye700DX-NHSester
Total		42157901	4213191				

B.

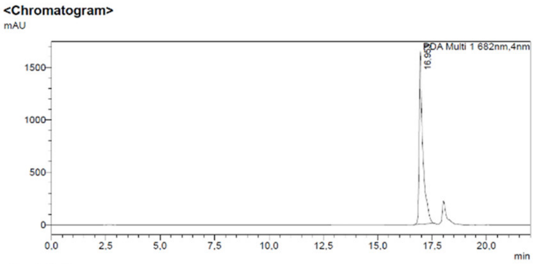

<Peak Table>

PDA Ch1 682nm

Peak#	Ret. Time	Area	Height	Conc.	Unit	Mark	Name
1	16,953	18801227	1648796	0,000	mg/L	M	IRDye700DX-COOH
Total		18801227	1648796				

Figure S4. **HPLC analysis of the hydrolysis reaction of IRDye700DX-NHS ester.** (**A**) Chromatogram of IRDye700DX-NHS ester. (**B**) Chromatogram after 1-week incubation at RT shows full hydrolysis of the IRdye700DX-NHS ester to IRdye700DX carboxylate. The chromatogram was recorded at 682 nm.

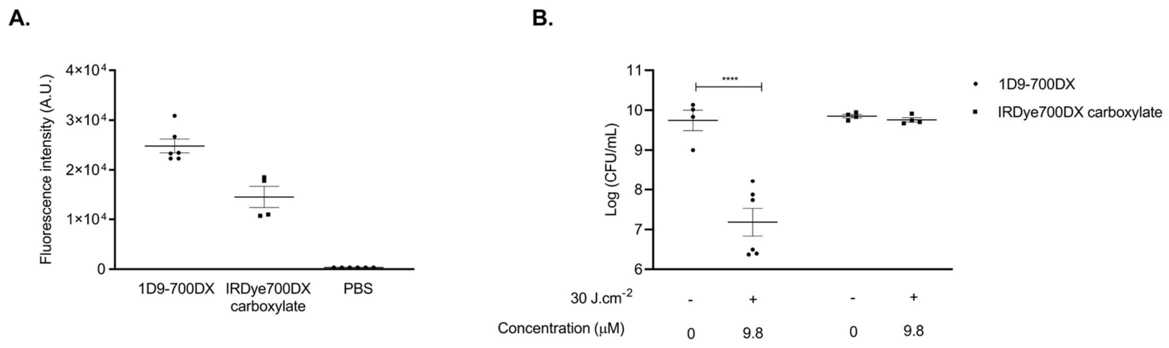

Figure S5. **Light-induced killing of *S. aureus* by 1D9-700DX and IRdye700DX carboxylate.** *S. aureus* AH4807 (MRSA) was grown to exponential phase (~10^9 CFU/mL) and incubated with 9.8 µM 1D9-700DX or IRDye700DX carboxylate with subsequent wash of the unbound compound with PBS. (**A**) Binding of 1D9-700DX or IRDye700DX carboxylate to *S. aureus* was assessed by fluorescence quantification measured in a Typhoon Biomolecular Imager and images were analyzed with Image J. (**B**) Samples from A were afterwards irradiated with 30 J.cm-2 of red light (+). Control samples were kept in the dark (-). Data are the mean value ± SEM of two experiments performed in duplicates. Two-way ANOVA test and subsequently the Sidak multiple comparisons test were used for statistical analysis of bacterial viability in B. Significant differences are marked as follows: *, p<0.03; **, p<0.002; ***, p<0.0002; ****, p < 0.0001.

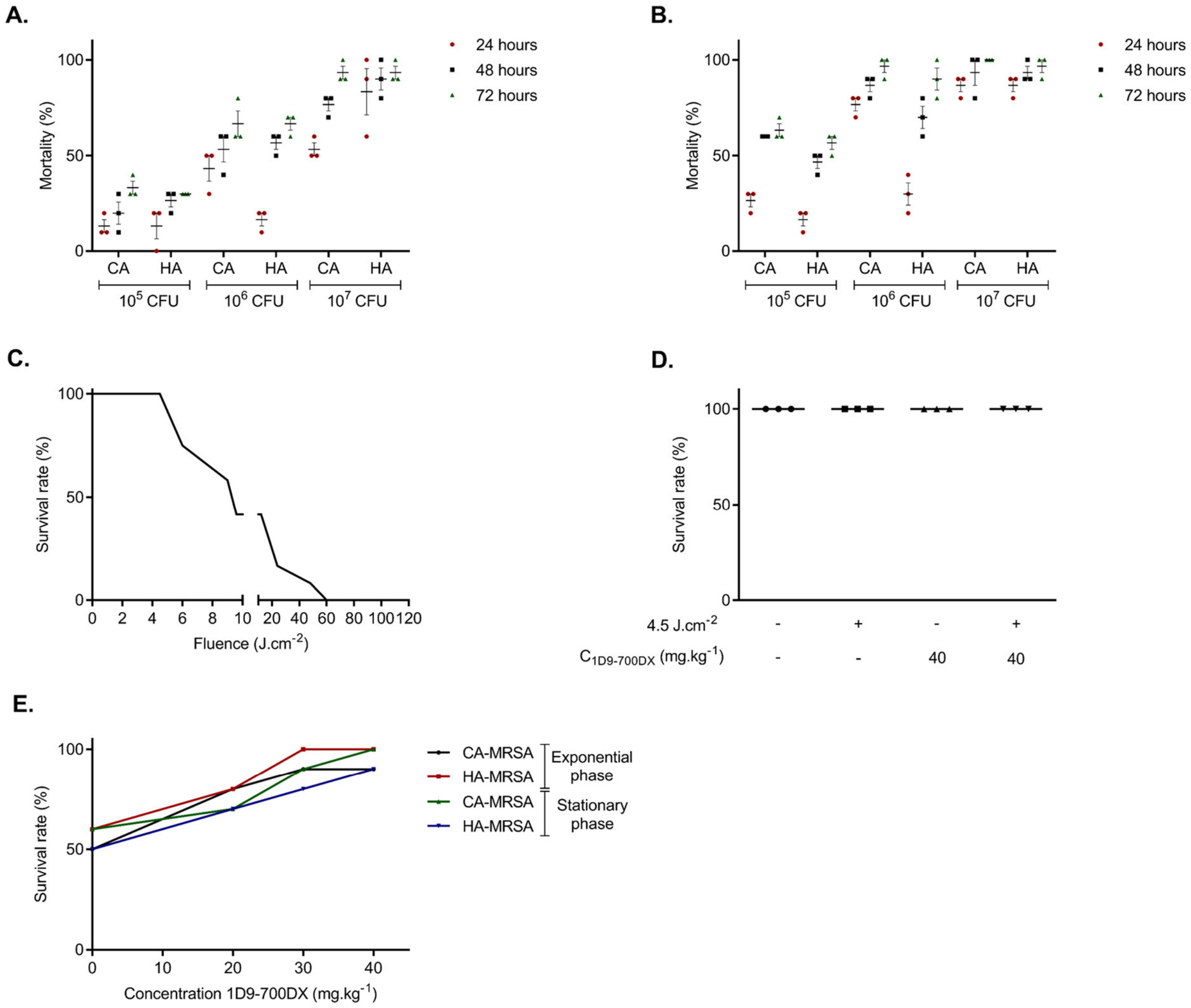

Figure S6. **Establishment of conditions for aPDT in the *G. mellonella* infection model.** (**A-B**) Comparison of the virulence of CA- and HA-MRSA strains in the *G. mellonella* model. Larvae were infected with *S. aureus* USA300 D15-GFP (CA-MRSA) or *S. aureus* USA300 D17-GFP (HA-MRSA) at doses of 10^5, 10^6 and 10^7 CFU/larva, using bacteria grown to the mid-exponential (**A**) or early-stationary (**B**) growth phases. Numbers of viable bacteria (CFU/ml) in the hemolymph of three surviving larvae were determined 24, 48 and 72 h post-infection. (**C**) Sensitivity of uninfected larvae to light. Larvae were exposed to 20, 100 or 200 mW.cm^{-2} of red light irradiation for 30, 45, 60, 240 or 600 seconds, and larval survival rates were assessed 72 h post-irradiation. (**D**) Effect of larval treatment with light (4.5 J.cm^{-2}) and 1D9-700DX (40 mg.kg^{-1}) alone, or in combination. A control with PBS and no irradiation was also included. (**E**) 1D9-700DX dose-response of aPDT in *G. mellonella* larvae infected with 10^6 CFU/larva of CA- or HA-MRSA grown to the mid-exponential or early-stationary growth phases. Survival rates were assessed 72 h post-irradiation. Data are the mean value ± SEM of three independent experiments with groups of 10 larvae (n=10).

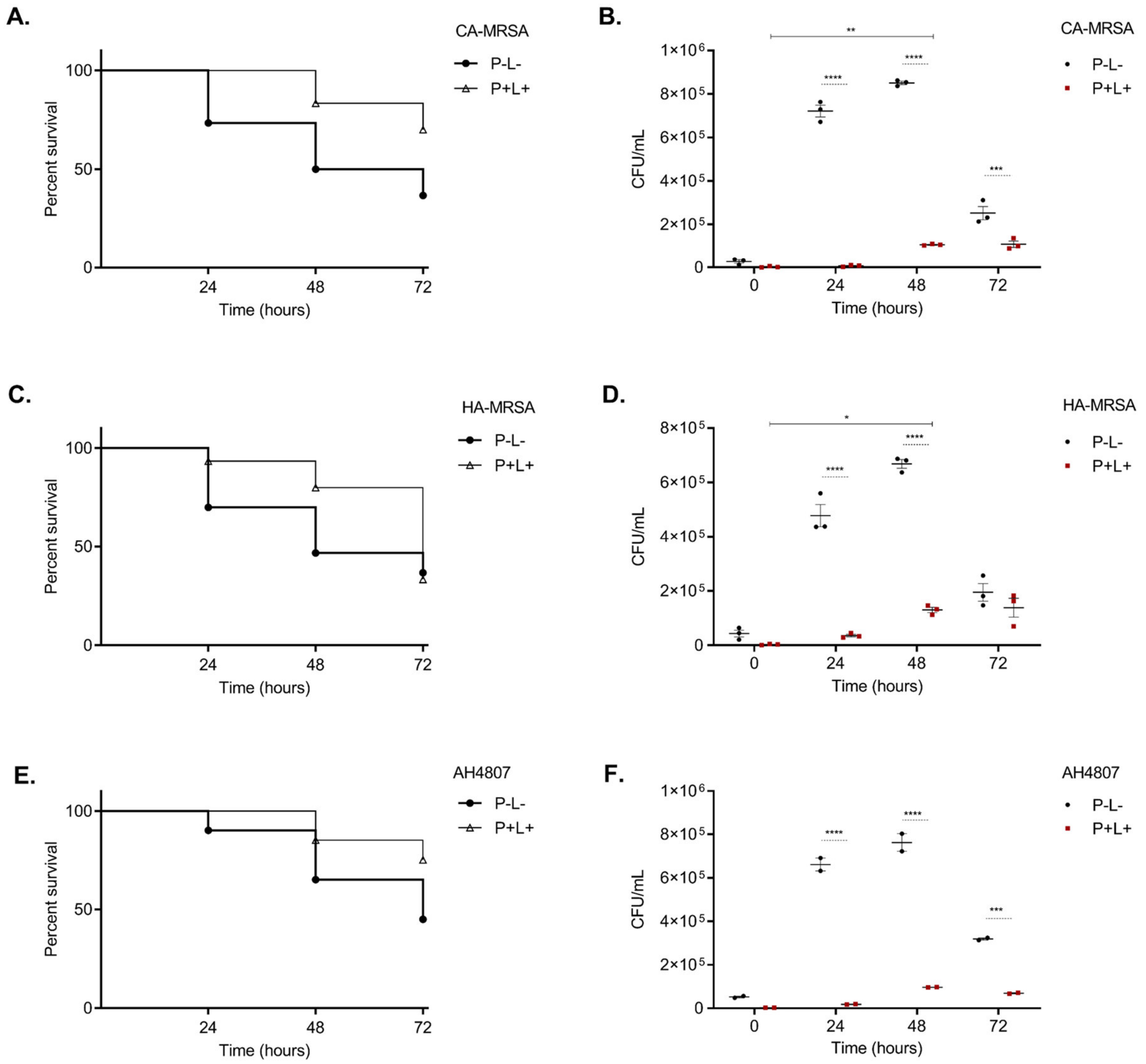

Figure S7. aPDT in the *G. mellonella* infection model using bacteria grown to early-stationary phase. Larvae were infected with CA-MRSA USA300 D15-GFP, HA-MRSA USA300 D17-GFP or CA-MRSA AH4807 grown to the early-stationary growth phase. For the P+L+ groups, 40 mg.kg^{-1} of 1D9-700DX (P+) were injected in the *G. mellonella* larvae at 90 min after bacterial inoculation. 30 min post-injection of the photosensitizer, light irradiation (L+) was performed at 4.5 J.cm^{-2} (45 seconds irradiation, 100 mW.cm^{-2}). The P-L- group neither received the immunoconjugate nor light exposure. (**A**, **C** and **E**) *G. mellonella* survival was monitored at 24, 48 and 72 h post-treatment. (**B**, **D** and **F**) Persistence of *S. aureus* in the hemolymph. Bacterial quantification (CFU/ml) in the hemolymph of three surviving larvae was determined at 0, 24, 48 and 72 h post-treatment. Data are the mean value ± SEM of three independent experiments with groups of 10 larvae (n=10). Gehan-Breslow-Wilcoxon and two-way ANOVA tests with subsequent Sidak multiple comparisons tests were used for statistical analysis of survival curves and bacterial persistence in the larvae hemolymph, respectively. Significant differences compared to the negative control group (P-L-) are marked as follows: *, p<0.03; **, p<0.002; ***, p<0.0002; ****, p < 0.0001.

Table S1: Bacterial strains used in this study.

Strain	Phenotype or Genotype	Source or Reference
***S. aureus* SH1000**	NCTC 8178 derived laboratory strain	(5)
***S. aureus* MS001**	SH1000 *isaA*::*tet* Tc^r	(6)
***S. aureus* Xen-36**	Wright (ATCC 49525) + *luxABCDE* (on a native plasmid)	(7)
***S. aureus* AH4807**	Community-acquired MRSA LAC(AH1263) *int*::*luxCDABEG*	(8)
***S. aureus* NCTC8325-4**	NCTC8325 cured of φ11, φ12, and φ13	(9)
***S. aureus* Newman**	NCTC 8178 derived laboratory strain	(10)
S. aureus* Newman Δ*spA*, Δ*sbi	*S. aureus* Newman *spA sbi* mutant	(11)
***S. aureus* clinical isolate**	Clinical isolate	UMCG
Staphylococcus epidermidis (ATCC® 35984™)	Catheter sepsis isolate	ATCC®
***S. aureus* CA-USA300 D15-GFP**	Community-acquired MRSA isolate expressing GFP	(12), this study
***S. aureus* HA-USA300 D17-GFP**	Hospital-acquired MRSA isolate expressing GFP	(12), this study
Escherichia coli (ATCC® 25922™)	*E. coli* type strain	ATCC®

Video S1. Three-dimensional reconstructions from stacks of two-dimensional confocal microscopy images of *S. aureus* NCTC8325-4 biofilm stained with the BacLight Live/Dead stain. The biofilm was incubated with 15.5 µM of 1D9-700DX and treated with red light LEDs at a radiant exposure of 30 $J.cm^{-2}$. Green fluorescence (Syto9) marks living bacteria, and red fluorescence (propidium iodide) marks dead bacteria. Scale bar: 45 µm.

References

1. van den Berg S, Bonarius HPJ, van Kessel KPM, Elsinga GS, Kooi N, Westra H, Bosma T, van der Kooi-Pol MM, Koedijk DGAM, Groen H, van Dijl JM, Buist G, Bakker-Woudenberg IAJM. A human monoclonal antibody targeting the conserved staphylococcal antigen IsaA protects mice against *Staphylococcus aureus* bacteremia. *Int J Med Microbiol.* 2015; 305(1):55–64.
2. Romero Pastrana F, Neef J, Koedijk DGAM, De Graaf D, Duipmans J, Jonkman MF, Engelmann S, Van Dijl JM, Buist G. Human antibody responses against non-covalently cell wall-bound *Staphylococcus aureus* proteins. *Sci Rep.* 2018; 8:3234.
3. de Boer E, Warram JM, Hartmans E, Bremer PJ, Bijl B, Crane LMA, Nagengast WB, Rosenthal EL, van Dam GM. A Standardized Light-Emitting Diode Device for Photoimmunotherapy. *J Nucl Med.* 2014; 55(11):1893–1898.
4. Zhao X, Medina LMP, Stobernack T, Glasner C, De Jong A, Utari P, Setroikromo R, Quax WJ, Otto A, Dö Rte Becher, Buist G, Van Dijl JM. Exoproteome Heterogeneity among Closely Related *Staphylococcus aureus* t437 Isolates and Possible Implications for Virulence. *J Proteome Res.* 2019; 18(7):2859–2874.
5. Horsburgh MJ, Aish JL, White IJ, Shaw L, Lithgow JK, Foster SJ. sigmaB modulates virulence determinant expression and stress resistance: characterization of a functional *rsbU* strain derived from *Staphylococcus aureus* 8325-4. *J Bacteriol.* 2002; 184(19):5457–5467.
6. Stapleton MR, Horsburgh MJ, Hayhurst EJ, Wright L, Jonsson IM, Tarkowski A, Kokai-Kun JF, Mond JJ, Foster SJ. Characterization of IsaA and SceD, two putative lytic transglycosylases of *Staphylococcus aureus*. *J Bacteriol.* 2007; 189(20):7316–7325.
7. Brand AM, De Kwaadsteniet M, Dicks LMT. The ability of nisin F to control *Staphylococcus aureus* infection in the peritoneal cavity, as studied in mice. *Lett Appl Microbiol.* 2010; 51(6):645–649.
8. Miller R, Crosby HA, Schilcher K, Wang Y, Ortines R V., Mazhar M, Dikeman DA, Pinsker BL, Brown ID, Joyce DP, Zhang J, Archer NK, Liu H, Alphonse MP, Czupryna J, Anderson WR, Bernthal NM, Fortuno-Miranda L, Bulte JWM, Francis, KP, Horswill AR, Miller LS. Development of a *Staphylococcus aureus* reporter strain with click beetle red luciferase for enhanced in vivo imaging of experimental bacteremia and mixed infections. *Sci Rep.* 2019; 9:16663.
9. Kreiswirth BN, Löfdahl S, Betley MJ, O'Reilly M, Schlievert PM, Bergdoll MS, Novick RP. The toxic shock syndrome exotoxin structural gene is not detectably transmitted by a prophage. *Nature.* 1983; 305:709–712.
10. Lorenz LL, Duthie ES. Staphylococcal coagulase; mode of action and antigenicity. *J Gen Microbiol.* 1952; 6(1-2):95–107.
11. Sibbald MJJB, Winter T, Van Der Kooi-Pol MM, Buist G, Tsompanidou E, Bosma T, Schäfer T, Ohlsen K, Hecker M, Antelmann H, Engelmann S, van Dijl JM. Synthetic effects of secG and secY2 mutations on exoproteome biogenesis in *Staphylococcus aureus. J Bacteriol.* 2010; 192(14):3788–800.
12. Mekonnen SA, Palma Medina LM, Michalik S, Loreti MG, Gesell Salazar M, van Dijl JM, Völker U. Metabolic niche adaptation of community- and hospital-associated methicillin-resistant *Staphylococcus aureus*. *J Proteomics.* 2019; 193:154–161.
